# Direct observation of mobility state transitions in RNA trajectories by sensitive single molecule feedback tracking

**DOI:** 10.1093/nar/gku1194

**Published:** 2014-11-20

**Authors:** Jan-Hendrik Spille, Tim P. Kaminski, Katharina Scherer, Jennifer S. Rinne, Alexander Heckel, Ulrich Kubitscheck

**Affiliations:** 1 Institute of Physical and Theoretical Chemistry, Rheinische Friedrich-Wilhelms-University Bonn, 53115 Bonn, Germany; 2 Institute for Organic Chemistry and Chemical Biology, Buchmann Institute for Molecular Life Sciences, Goethe University Frankfurt, Max-von-Laue-Str. 9, 60438 Frankfurt, Germany

## Abstract

Observation and tracking of fluorescently labeled molecules and particles in living cells reveals detailed information about intracellular processes on the molecular level. Whereas light microscopic particle observation is usually limited to two-dimensional projections of short trajectory segments, we report here image-based real-time three-dimensional single particle tracking in an active feedback loop with single molecule sensitivity. We tracked particles carrying only 1–3 fluorophores deep inside living tissue with high spatio-temporal resolution. Using this approach, we succeeded to acquire trajectories containing several hundred localizations. We present statistical methods to find significant deviations from random Brownian motion in such trajectories. The analysis allowed us to directly observe transitions in the mobility of ribosomal (r)RNA and Balbiani ring (BR) messenger (m)RNA particles in living *Chironomus tentans* salivary gland cell nuclei. We found that BR mRNA particles displayed phases of reduced mobility, while rRNA particles showed distinct binding events in and near nucleoli.

## INTRODUCTION

Different aspects of molecular mobility in living specimen can be probed by a plethora of fluorescence microscopy techniques (e.g. fluorescence recovery after photobleaching (FRAP) (1), fluorescence correlation spectroscopy (FCS) (2), single pair fluorescence resonance energy transfer (spFRET) (3), single particle tracking (SPT) (4)). The observation of single particle dynamics has yielded important insight into plasma membrane organization (5), virus infection mechanisms (6) and has even enabled direct observation of the walking steps of motor proteins (7). However, trajectories are often limited to a low number of localizations by the small depth of field (< 1 μm) of high numerical aperture (NA) detection objectives (8,9). Elaborate statistical methods are required to identify mobility states and state transitions in large ensembles of short trajectories (8). Also, an anisotropic specimen structure within the axial detection range can introduce artifacts, if two-dimensional (2D) projections of three-dimensional (3D) particle motion are analyzed (10).

Full spatial information for an individual particle can be obtained by orbital scanning of a point detector and simultaneous widefield detection (11). Various image-based methods yield 3D localizations for all particles in the detection volume, e.g. by observing multiple focal planes simultaneously (12,13), interferometric detection (14) or point spread function (PSF) engineering (15,16). However, axial localization ranges remain limited to 1–2 μm. In orbital scanning, the axial range of trajectories is extended by incorporating a feedback mechanism, which constantly centers the detection volume on the position of a single particle. Similarly, feedback tracking was also demonstrated for bi-plane detection of a small image field (17) and astigmatic detection (18). Nevertheless, the limited number of photons available from single fluorophores hitherto precluded the observation of trajectories substantially exceeding the mentioned axial range (11,13,17–18). Trapping approaches with single fluorophore sensitivity have been used to detect interaction kinetics in solution on a single molecule level but interfere with particle motion and are thus not suited for studies of molecular trafficking *in vivo* (19). To overcome this limitation, we have developed a method enabling 3D localization and feedback tracking with several hundred localizations per trajectory based on only 100–400 photons detected per frame. Its improved sensitivity finally extends the scope of feedback tracking to the observation of biomolecules labeled with single dye molecules *in vivo*.

In our experiments, light sheet microscopy provided intrinsic optical sectioning and enabled single particle observation with exceptionally high signal-to-noise ratio (SNR) (20). Astigmatic detection was used to encode 3D localizations in the shape of the PSF (Supplementary Figures S1 and S2). In order to extract this information on-the-fly and with high precision, we devised a novel axial localization metric based on cross-correlation template matching. 3D coordinates were determined rapidly in < 200 μs (Supplementary Figure S3) and the axial sample stage position adjusted in an active feedback loop. Thus, the focal plane of the instrument followed the axial position of the particle of interest. At a given time, the real-time algorithm tracked only a single particle in its current implementation. Nevertheless, full image data were acquired. During post-processing, all particles in the detection volume were tracked and absolute 3D trajectories reconstructed by taking the respective sample stage position into account. Since trajectories were no longer limited by the axial detection range of the instrument but rather by the number of photons emitted before bleaching of the label, hundreds of localizations could be recorded per particle.

We demonstrate the single fluorophore sensitivity of the method by following lipids labeled with a single organic dye molecule in the membrane of giant unilamellar vesicles (GUVs) with as little as 130 photons detected per signal. Trajectories of more than 1000 localizations were ultimately limited by the photon yield of the dye molecule and spanned 5–10 μm in all spatial dimensions. Observation times longer than 20 s were achieved at a frame rate of 60 Hz, more than one order of magnitude longer than without feedback tracking. We further employed the method for intranuclear tracking of messenger RNA (mRNA) and ribosomal RNA (rRNA) particles more than 100 μm deep inside living tissue. Particles were labeled with specific antisense oligonucleotides carrying small synthetic dyes to minimize artifacts due to the size of the label. Feedback tracking increased the number of localizations per trajectory by an order of magnitude or more. The high number of localizations allowed for a rigorous statistical analysis of the temporal sequence of jump distances in individual trajectories. We were thus able to directly observe repeated transitions between mobility states for single particles, identify dwell times in states of reduced mobility and quantify their significance (21).

## MATERIALS AND METHODS

### Instrumentation and feedback loop

Our light sheet microscope was built around a commercial inverted microscope (TI-Eclipse, Nikon, Duesseldorf, Germany) as previously described (18). A thin sheet of light (2.2 μm full width at half-maximum beam waist) was focused into a custom-made glass sample chamber (105-044-V2-40, Hellma, Muellheim, Germany). Fluorescence was collected through the coverslip bottom by a long working distance water immersion objective (40× CFI Apo LWD S, NA 1.15, Nikon). A weak cylindrical lens (LJ1516RM, Thorlabs, Dachau, Germany) was placed in the detection path between tube lens and a 2.5× additional magnification lens (VM2.5×, Nikon), close to the primary image plane, to introduce astigmatism for 3D localization. Images were recorded with either an EMCCD (Ixon DU860/DV897, Andor, Belfast, UK) or sCMOS camera (pco.edge, PCO, Kelheim, Germany). Experiments were performed using ImSpector 5.5 instrument control software (LaVision BioTec, Bielefeld, Germany) extended by the TrackDev module enabling axial sample positioning by a piezo stage (P-611.ZS with E-625 controller, Physik Instrumente, Karlsruhe, Germany) controlled via analog output of a DAQ card (ME4660, Meilhaus Electronic, Alling, Germany). Axial positioning of the sample in a feedback loop was achieved by

1. encoding axial localizations relative to the focal plane in the shape of the PSF,
2. extracting the information by fast image analysis as soon as image data were transferred from the camera chip to PC memory and
3. converting the axial information into a voltage and applying it to the piezo stage.

### PSF measurements and calibration data sets

The PSF of the instrument was characterized by filling the sample cuvette with 200 nm fluorescent beads (TetraSpeck 0.2 μm, Invitrogen Life Technologies, Darmstadt, Germany) embedded in 1.5% agarose. The sample was subsequently moved through the focal plane in steps of 50 nm using the piezo stage, resulting in high SNR PSF images. Bead images were fitted by an elliptical Gaussian peak and the PSF width in *x* and *y* plotted versus *z*-position. Inserting the cylindrical lens resulted in a shift between the minima in *x* and *y* indicating the respective focal planes. An effective focal plane (*z*_rel_ = 0) was defined as the position of equal PSF width along the two dimensions. To obtain an axial localization calibration curve, two PSF templates (subimages of 9×9 to 11×11 pixels centered on the intensity peak) were extracted from the image stack at distances of *z*_rel_ = ± 400 nm from the effective focal plane. This corresponded to the approximate separation between *x* and *y* focal plane (the amount of astigmatism). For each slice of the calibration stack, normalized covariance images with the two templates were calculated according to Equation (10) in (21). For each slice, the two covariance values ξ_1_ and ξ_2_ for the pixel with the highest sum of both values were used for further calculations. Between the *x* and *y* focal plane, a linear calibration curve was found by plotting (ξ_2_ – ξ_1_) / (ξ_1_ + ξ_2_) as a function of axial position *z*_rel_ relative to the effective focal plane and fitting with

(1) \begin{equation*} \frac{{\xi_{2} - \xi_{1}}}{{\xi_{1} + \xi_{2}}} = a \cdot {z}_{{\rm rel}} + {b} \end{equation*}

to obtain the calibration parameters *a* and *b*. They depended on the amount of astigmatism as well as the template size, but not on SNR within the tested range of 4–65 (Supplementary Figure S5). Typical values for experiments presented here were SNR = 6–10.

### 3D localization during real-time tracking

Image analysis code for real-time tracking was written in C++ using Microsoft Visual C++ 2010 Express. It was incorporated in a dynamic link library (DLL) called by the ImSpector TrackDev module as soon as an image frame had been transferred to PC memory (Supplementary Figure S4). Commented source code and a manual are provided in Supplementary Material 1. Lateral particle coordinates were obtained by localizing an intensity maximum within a search radius around either user-supplied initial coordinates or a previous particle localization and subsequent centroid calculation (18). For axial localization, normalized covariance values with PSF templates were calculated in a neighborhood of 3×3 to 5×5 pixels around the intensity peak as described above. The two covariance values ξ_1_ and ξ_2_ for the pixel with the highest sum of both values were used for direct calculation of axial localizations from the inversed formulation of Equation (1)

(2) \begin{equation*} {z}_{{\rm rel}} = \left( {\frac{{\xi_{2} - \xi_{1}}}{{\xi_{1} + \xi_{2}}} - {b}} \right)/{a}. \end{equation*}

### Particle tracking during post-processing and trajectory analysis

During post-processing, all particle trajectories were recovered from the image data using MATLAB (The Mathworks, Natick, MA, USA) code. Particles were identified by cross-correlating images with PSF templates and finding local maxima above a threshold. A simple nearest neighbor approach with a user-supplied upper limit for displacements was sufficient for connecting localizations to trajectories since the concentration of labeled particles was extremely low, typically < 1 pM. In ambiguous situations, trajectories were terminated. Diffusion coefficients were determined from mean square displacement (MSD) plots, jump distance histograms (22) or cumulative jump distance distributions by fitting the cumulative probability function to the data:

(3) \begin{equation*} {P}({r}^2 ,\Delta {t}) = 1 - \sum\nolimits_i {{a}_{i} {e}^{ - {r}^2 /(4{D}_{i} \Delta {t})} }. \end{equation*}

In the case of 2D diffusion on the spherical GUV surface, 3D Euclidean displacements were considered and analyzed with 2D equations. At typical displacements <300 nm and a GUV radius of >20 μm, errors introduced by using the distance instead of the arc length of the displacement were negligible. 2D displacements (*xy*) were considered for *in vivo* tracking since, as in most 3D localization approaches, lateral localization precision was superior to axial localization and no evidence of an anisotropic behavior was found. (For dwell time analysis, trajectory segments were reviewed to verify that 2D confinements not simply represented phases of predominantly axial motion.)

### Dwell time analysis and detection of state transitions

A single particle trajectory comprises a number of jumps with squared distances *r*^2^. Setting a threshold *r^2^*_l__im_ assigns a state to each jump distance in any trajectory. If *P*(*r*^2^) denotes the cumulative jump distance distribution, *P*_A_ *= P*(*r*^2^_lim_) is the probability to find the particle in state A with *r*^2^ ≤ *r*^2^_lim_ at any given time point, whereas *p*_B_ = 1 − *p*_A_ applies for state B with *r*^2^ > *r*^2^_lim_. Thus, the temporal sequence of jump distances can be regarded as a series of Bernoulli trials with success probability *p*_A_ to find the particle in state A. Due to the stochastic nature of Brownian motion, the state of the particle will change frequently. The probability *P’*(*n*,*k*,*P*) to observe a dwell time of *k* frames in a state with probability *P* in a series of *n* jump distances can be calculated according to Equation (13) in (21):

(4) \begin{equation*} \begin{array}{*{20}l} {P'(n,k,P) = } \\ {\sum\nolimits_{m = 0}^{\frac{{n + 1}}{{k + 1}}} {( - 1){}^mp^{mk} (1 - p)^{m - 1} \left( {\left( {\begin{array}{*{20}c} {n - mk} \\ {m - 1} \\ \end{array}} \right) + (1 - p)\left( {\begin{array}{*{20}c} {n - mk} \\ m \\ \end{array}} \right)} \right)} } \\ \end{array}. \end{equation*}

The longer a dwell time in either state, the lower the probability to observe such an event in a sequence of jump distances randomly drawn from *P(r^2^)*. Low *P*-values with *P’*′ < 0.05 are regarded as significant deviation from random behavior. Generally, dwell times below a threshold *r*^2^_lim_ indicate the absence of large particle displacements, which could, in experimentally acquired trajectories, suggest a state of reduced particle mobility. Thus, we can compare *P’*(*n*,*k*,*P*) resulting from experimental observations to analytically determined expectation values for a random trajectory following the same jump distance distribution *P*(*r*^2^).

Similarly, the expected number of transitions between state A and B and vice versa in a series of *n* jump distances randomly drawn from *P*(*r*^2^) and its standard deviation are given by

(5) \begin{equation*} \langle s'\rangle = 2p_{\rm A} p_{\rm B} n \end{equation*}

(6) \begin{equation*} \sigma _{s'} = \sqrt {p_{\rm A} p_{\rm B} n}. \end{equation*}

The number *s* of transitions between states A and B can simply be counted in trajectories. If the experimentally determined value *s* is significantly lower than the expectation value <*s’>*, the particle under observation resides longer in either of the states than expected for random Brownian motion. Thus, such a deviation from the expectation value can be used to assess the presence of retention times on the timescale of the temporal resolution of the experiment, e.g. due to transient binding events, without the need to actually identify dwell times in such a state.

### Lipid labeling and GUV preparation

Cholesterol, 1,2-dihexadecanoyl-sn-glycero-3-phosphocholine (DPPC), 1,2-dipalmitoyl-sn-glycero-3-phosphoethanolamine (DPPE) and 1-hexadecanoyl-2-(9Z-octadecenoyl)-sn-glycero-3-phosphoethanolamine (POPE) were purchased (Avanti Polar Lipids, Alabaster, AL, USA) and used without further purification. Lipids were dissolved in chloroform (J.T. Baker, Deventer, the Netherlands) to a final concentration of 1.3 mM. ATTO647-NHS-ester (ATTO-TEC, Siegen, Germany), methanol (Sigma Aldrich, Hamburg, Germany) and triethylamine (NEt3, Carl Roth, Karlsruhe, Germany) were used for lipid labeling reactions. Lipids were fluorescently labeled by coupling ATTO647-NHS-ester to the amino group of the phospholipids DPPE and POPE. The reaction was carried out with an equimolar amount of dye in chloroform/methanol (2:1 volume ratio) for 1 h at room temperature upon addition of 15 molar equivalents of NEt3 (23). Labeled lipids were purified by thin layer chromatography and extracted with methanol.

GUVs were created by electro-formation (24,25) in 250 mM sucrose solution as described previously (26). Vesicles were prepared from mixtures of DPPC and cholesterol with a molar ratio of 9:1 and 1:1. Fluorescent lipids were included with a fraction of 10^−6^ to 10^−7^ mol% for single particle tracking. For measurements, GUVs were diluted in 250 mM glucose solution. Because of the density difference between the sugar solutions GUVs deposited and resided stably at the chamber bottom.

### Oligonucleotide synthesis and labeling

The BR2.1 mRNA oligonucleotide carrying a single Atto647N dye molecule at the 5′ end was purchased from IBA (IBA GmbH, Göttingen, Germany). All other oligonucleotides were synthesized on an Applied Biosystems Model 392 synthesizer (ABI Life Technologies) using 2′-OMe-RNA phosphoramidites. All standard cyanoethyl phosphoramidites were purchased from Link Technologies Ltd. and employed according to recommended coupling protocols. For internal amino-group modification a commercially available amino-modifier C6-dT-CE phosphoramidite (Link Technologies Ltd., Bellshill, UK) was inserted at the respective position. The 3′-amino modification was introduced with a 3′-amino-modified solid phase ((CH_2_)_7_-linker, CPG 1000 Å, 200 nmol). An additional 5′-amino modifier was introduced using a 5′-monomethoxy trityl-amino-modifier C6-CE phosphoramidite. The terminal monomethoxy trityl group was removed at the synthesizer.

After synthesis the oligonucleotides were cleaved from the solid support and deprotected using ammonia solution (32% (v/v)) at 65°C for 2 h. Subsequently, oligonucleotides were purified via reversed-phase high performance liquid chromatography (HPLC). Nucleosil 100-5 C18 columns were used with a gradient of 0.1 M triethylammonium acetate (pH 7) and acetonitrile. The percentage of acetonitrile was increased from 0% to 47% within 50 min.

Oligonucleotide identity and successful dye labeling were confirmed by electrospray ionization mass spectrometry (Supplementary Table S2). Microinjection of bulk quantities of the labeled oligonucleotides into *Chironomus tentans* salivary gland cell nuclei demonstrated the functionality of the probes. In case of the BR2.1 oligo-3xAtto647N, a distinct labeling of the Balbiani ring (BR) transcription sites was achieved; in case of the 28S rRNA oligo-3xAtto647 the nucleoli of respectively injected nuclei were clearly labeled.

### Postsynthetic dye labeling

Atto647 and Atto647N were purchased as reactive N-hydroxysuccinimide (NHS)-esters (ATTO-TEC). Labeling of the amino-modified oligonucleotide with Atto647N was performed according to standard NHS-labeling conditions using 0.1 M NaHCO_3_ buffer (pH 8.5) for 90 min in the dark. For the Atto647-dye, basic pH values are known to be a critical issue due to degradation of the dye according to the manufacturer. Therefore, the reaction was performed at pH 7.9–8.0 for 120 min in the dark. Generally, 1.5 equivalents of the respective dye were used compared to the available aliphatic amino groups within the oligonucleotide. Afterward, the reaction mixtures were purified via reversed-phase HPLC using the same conditions as described above. The identity and labeling of the 28S rRNA and BR2 oligonucleotides were verified by mass spectrometry. Each oligonucleotide was labeled by three dye molecules (Supplementary Table S2).

### *C. tentans* sample preparation

Preparation of *C. tentans* salivary glands and microinjection as well as NTF2 expression and labeling were described in detail in a previous publication (27). Briefly, salivary glands were explanted in phosphate buffered saline (PBS) and transferred into a poly-L-Lysine coated sample chamber. Microinjection was performed with 70–90 hPa holding and 500–1000 hPa injection pressure for 0.1–0.3 s using the manual injection mode and a 40x water immersion objective lens. PBS was used as buffer for the injection solution. Injection solutions were prepared from stock for each experiment and contained the respective oligonucleotides in a concentration of 25 nM and NTF2-Alexa546 in a concentration of up to 50 μM. For the mRNP tracking experiments, Sytox Green (Invitrogen) was delivered separately by an additional injection.

### Image acquisition

Single particle tracking data were acquired with kinetic cycle times as stated in the main text. EMCCD cameras were used in frame transfer mode. Red dyes used for single particle tracking were excited with a 640 nm laser line (Cube 640–40C, Coherent, Santa Clara, CA, USA). Reference image stacks were acquired by displacing the piezo stage in steps of 500 nm while exciting SYTOX Green with a 488 nm laser line (Sapphire-100, Coherent) or NTF2-AlexaFluor546 with a 532 nm laser (LasNova GLK, Lasos, Jena, Germany). The astigmatic lens was left in place during acquisition of reference images. Tracking images were overlaid with reference stack images according to the piezo stage voltage registered for the respective image frames. Appropriate notch filters for the respective experiments (NF01–488/532/640U-23.7-D, Semrock IDEX, Lake Forest, IL, USA) were placed in the detection arm.

## RESULTS

### Feedback tracking at low photon count

**Figure 1.**
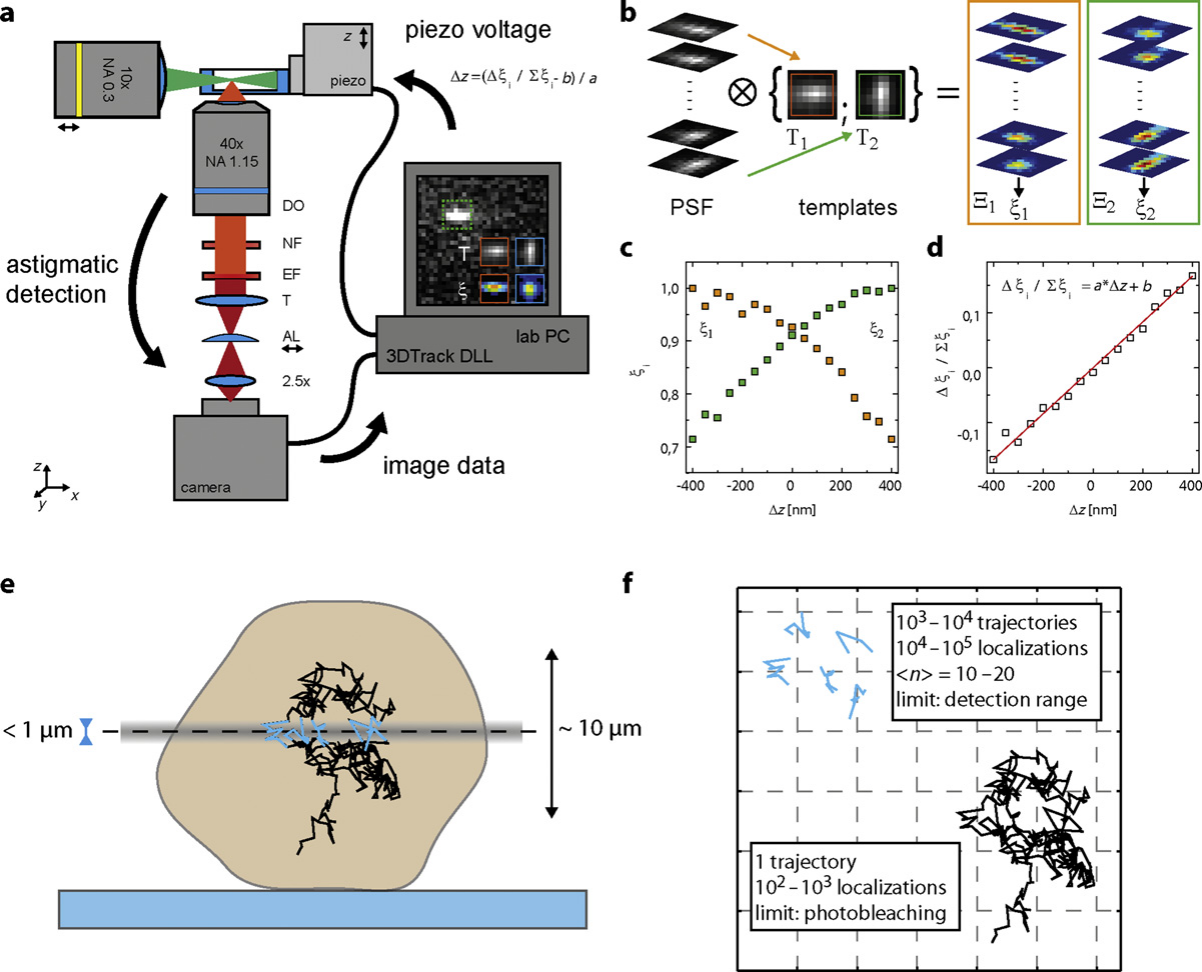
Schematic of the 3D feedback tracking setup. (**a**) Fluorescence was excited by light sheet illumination and detected by a high NA water immersion objective. Information on 3D particle positions was encoded by astigmatic imaging. Image data were analyzed ‘on-the-fly’ (Supplementary Figure S4) and particle localizations determined in real-time (Supplementary Figure S3). The positional information was used to drive a piezo stage and keep a particle of interest close to the focal plane. DO: detection objective, NF: notch filter, EF: emission filter, T: tube lens, AL: astigmatic cylindrical lens, 2.5×: additional magnification lens. (**b**) Feedback tracking at low photon counts was achieved by using template matching for axial localization. Two templates T_1,2_ were extracted from a PSF stack, and used to calculate normalized covariance images Ξ_1,2_ with an unknown signal. (**c**) Normalized covariance values ξ_1_ and ξ_2_ extracted from the pixel with the maximum sum of Ξ_1_ and Ξ_2_ can serve as unambiguous axial calibration curves. (**d**) A precise linear metric for axial particle localization independent of SNR can be found (Supplementary Figure S5). (**e**) The confined axial detection range (gray) usually restricts the duration of trajectories (blue). Feedback tracking can extend the observation time per particle up to the limit of photobleaching. (**f**) While short observations require ensembles of thousands of trajectory snippets to be analyzed, feedback tracking facilitates true single particle studies.

Key to feedback tracking with a low number of photons per localization is a photon-efficient instrument and a sensitive detection algorithm. We collect fluorescence excited by light sheet illumination with a high NA objective and detect it with an EMCCD or sCMOS camera. Labeling strategies were optimized to achieve sparse, highly specific single molecule signals with low unspecific background. Astigmatic detection was chosen for 3D localization because it provides full spatial information for all particles in an image frame and neither divides the precious fluorescence photons between multiple image planes nor requires their computational registration. The feedback loop for active particle tracking consists of a DLL coupled to the instrument control software extracting positional information from the image data as soon as they have been transferred from the camera chip to PC memory (Supplementary Figure S4, Supplementary Material 1), and a piezo stage, which is used to reposition the entire sample holder based on the DLL output. Thus, a particle of interest can continuously be kept within the light sheet and close to the focal plane (Figure 1).

Previously, we used a first and second moment calculation to rapidly extract an axial localization from the shape of the PSF (18). However, this approach required a high number of photons per localization, leading to fast bleaching of fluorescent labels and inaccurate results at low photon numbers (28). The width of an elliptical Gaussian peak approximated to the intensity distribution yields an accurate measure of the PSF shape even at low SNR (29). Alas, this approach involves an iterative least squares optimization, which took more than 1 ms per localization on our system with exact durations depending on the number of iterations before convergence of the algorithm. Lower SNR generally required a higher number of iterations. To overcome these limitations, we developed an axial localization metric based on the cross-correlation values between a particle image and two templates representing the PSF shape at a distance above and below the focal plane. While using an experimentally acquired PSF for particle localization has been reported before (30,31), our approach requires only two templates extracted from a calibration data set for accurate 3D localization. A precision equal to least square minimization but with 7–15× faster calculation was achieved for this approach (Supplementary Figure S3). Further, we found a normalized metric independent of SNR and directly proportional to the axial position of an emitter such that only two parameters, the slope and the offset of a calibration curve, are sufficient for axial localization (Supplementary Figure S5). We achieved an overall temporal resolution down to 1.1 ms (frame rate 892 Hz), while tracking fluorescent beads in aqueous solution (Supplementary Figures S6 and S7, Supplementary Video S1) enabling feedback tracking of particles with a diffusion coefficient of up to 10 μm^2^/s (Supplementary Figure S8), corresponding to the intracellular mobility of a fluorescent protein (32).

### Single trajectory analysis

**Figure 2.**
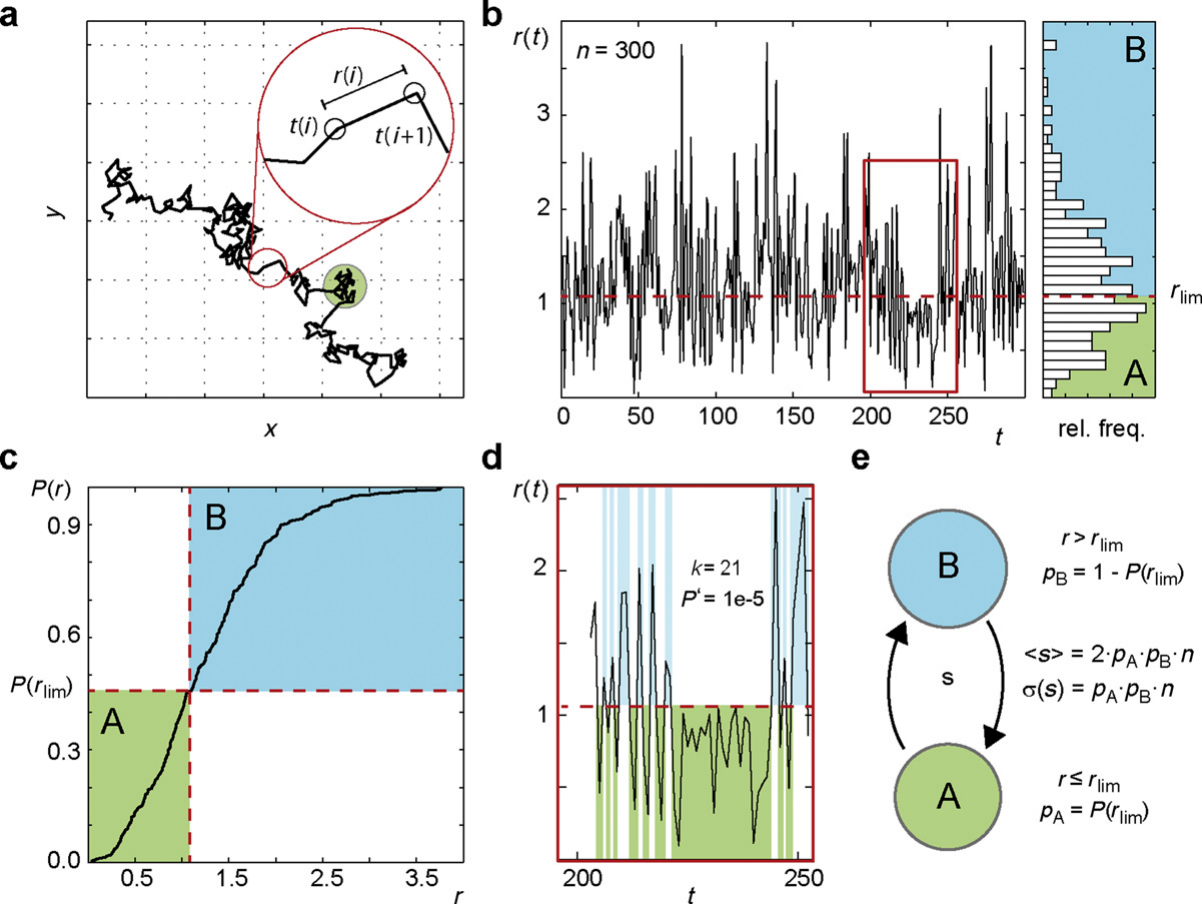
Trajectory analysis. (**a**) Simulated trajectory consisting of 301 time points *t*(*i*) and 300 displacements *r*(*i*). (**b**) Temporal sequence of displacements obtained from the trajectory (left panel) and jump distance distribution (right panel). Selecting a threshold *r*_lim_ defines states A with *r*(*i*) ≤ *r*_lim_ and B with *r* > *r*_lim_. (**c**) From the cumulative jump distance distribution *P*(*r*), the relative frequency of both states can be inferred. (**d**) Within the trajectory, particularly long dwell times in either state can be identified and their significance *P’*′ quantified according to (Equation (4)). (**e**) The existence of distinct mobility states can be further substantiated by differences between the transitioning rate *s* between A and B and the expectation value *<s>* (Equation (5)).

If not limited to the quasi-planar surface of a cell membrane, typical intracellular trajectories of mobile particles contain no more than 10–20 localizations limited by the small axial detection range. Feedback tracking can yield hundreds of localizations in a single trajectory. In addition to the probability distribution of jump distances in an ensemble of trajectories (33), their temporal sequence in individual trajectories can now be taken into account (Figure 2). Due to the stochastic nature of free particle diffusion, the length of particle displacements varies randomly between small and large values drawn from a characteristic probability density function. The trajectory of a particle transiently interacting with a larger particle or cellular compartment is characterized by the absence of large displacements during the interaction time. The high number of localizations per particle achieved by feedback tracking allows a probability (*P*’-value, Equation (4)) to be assigned to such phases or dwell times, describing the probability to observe the sequence of particle displacements if they were drawn randomly from the experimentally observed jump distance distribution. Thus, the significance of dwell times in a state of reduced mobility can be stated in a quantitative, unbiased manner. This aspect of the molecular motion allows for a deeper understanding of the kinetics underlying the jump distance distribution and directly reveals transitions between states of different mobility (Figure 2c–e) whereas an ensemble analysis merely yields relative frequencies of mobility states.

### Tracking lipids carrying single fluorophores

**Figure 3.**
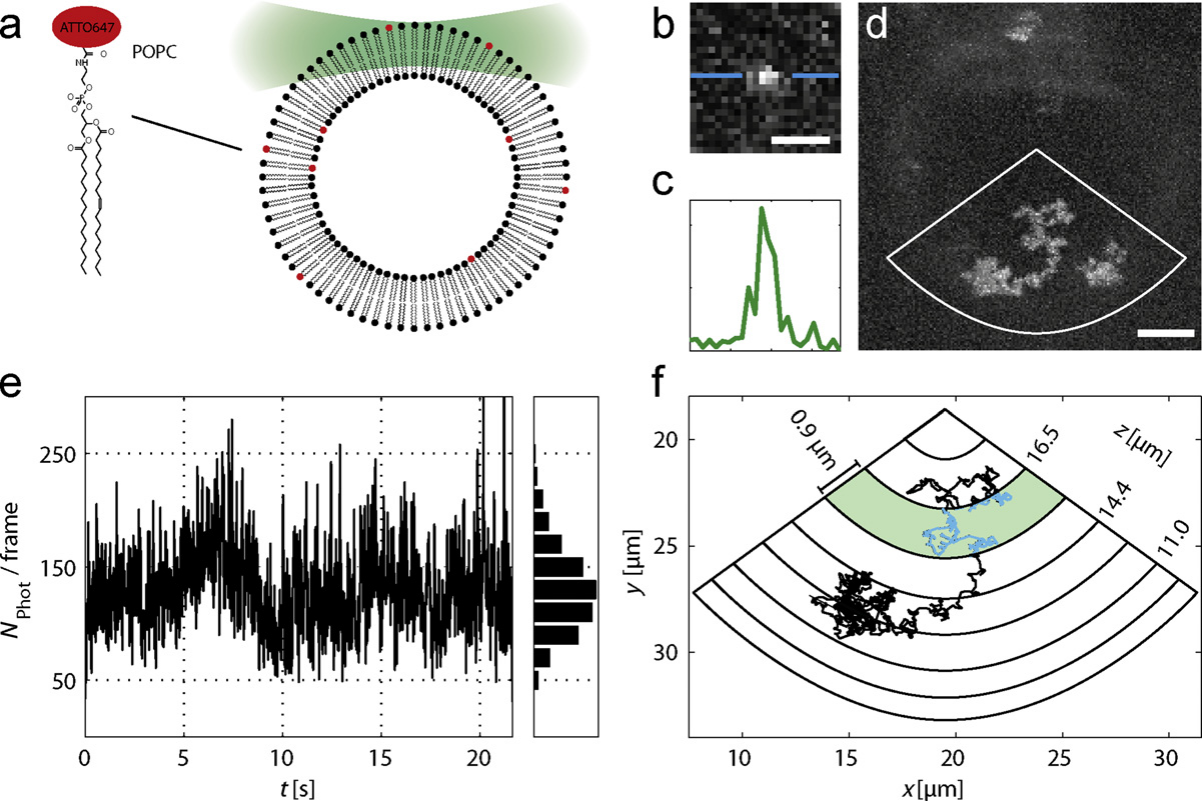
Single lipid tracking in GUV membranes (sketch not to scale). (**a**) POPE lipids were labeled with single ATTO647 dye molecules (Supplementary Figure S9) and incorporated at ∼10–7 mol% into GUVs consisting of DPPC and cholesterol at a molar ratio of 1:1. (**b**) Raw image of a fluorescent lipid molecule extracted from one frame of the time series (scale bar, 1.5 μm). (**c**) Line profile through the intensity peak as indicated in (b). (**d**) Maximum intensity projection of 1354 image frames acquired at different axial positions throughout the course of an exemplary trajectory (Supplementary Video S2). Scale bar, 5 μm. (**e**) Detected photons as a function of time. On average, only 130 photons were detected per signal, adding up to a total of 1.76 × 10^5^ photons detected from the molecule. (**f**) Polar plot representation of the trajectory. The green area highlights the approximate depth of focus of the detection objective and thus the axial detection range, which would have been achieved without feedback tracking.

To demonstrate the sensitivity and robustness of our technique, we inserted DPPE lipids labeled by a single ATTO647 dye into the membrane of a common model system for biological membranes, GUVs consisting of DPPC and cholesterol (1:1). Single emitter labeling was confirmed by single step photobleaching of immobilized DPPE-ATTO647 in membranes of higher DPPC to cholesterol ratio (9:1) (Supplementary Figure S9). Trajectories of more than 1300 localizations covering a large fraction of the spherical vesicle surface could be recorded with our tracking approach. Individual trajectories spanned up to 8 μm not only in lateral but also axial direction (Figure 3). A total observation time of more than 20 s at a temporal resolution of 16.5 ms was achieved for individual lipids (Supplementary Video S2). Due to the optical sectioning effect of light sheet microscopy, the background noise could be reduced to < 2 photons/pixel (s.d.). This exceptionally low value enabled feedback tracking at as little as 130 photons detected per signal. As expected, random diffusion of the particle in the lipid membrane was observed. A fit to the MSD yielded a diffusion coefficient of *D* = 0.82 ± 0.02 μm^2^/s.

### *In vivo* tracking of BR2.1 mRNA particles

A tremendous prolongation of trajectory duration was also achieved for tracking of biomolecules in living tissue. Fluorescently labeled oligonucleotides directed against BR2.1 mRNA were microinjected into *C. tentans* salivary gland cell nuclei. Either the fluorescently labeled nuclear transport factor NTF2-Alexa546 or DNA-staining SYTOX Green or both were co-injected for imaging with up to three fluorescence channels. NTF2-Alexa546 highlighted the nucleoplasm and resulted in a rim staining of the nuclear envelope. Exclusion of NTF2-Alexa546 from polytene chromosomes and nucleoli enabled identification of these nuclear compartments as dark areas within the nucleus. Light sheet fluorescence microscopy facilitated rapid acquisition of reference image stacks showing the nuclear envelope and DNA signal. Since only mild image distortions were introduced by the astigmatic PSF (Supplementary Figure S1), reference images of the specimen could be acquired with the same detection channel and superimposed with single particle image data without further image registration. This allowed us to relate single molecule trajectories to the surrounding structure of the specimen and supported the interpretation of the observed single molecule dynamics.

Trajectories covering an axial range of several microns were readily acquired from BR2.1 mRNA particles labeled with oligonucleotides carrying a single Atto647N dye molecule (Supplementary Figure S10, Supplementary Video S3). However, background noise more than 100 μm deep within the tissue was increased three-fold or more as compared to the lipid tracking experiments and photobleaching limited trajectory duration. To meet this challenge and achieve a further prolongation of trajectories we modified the oligonucleotides to carry three dye molecules (Supplementary Table S2).

A classical single particle trajectory ensemble consisting of 21 533 displacements in 1243 trajectories with at least three localizations per trajectory was obtained and divided into two spatially separated subsets. The first contained trajectories detected in the nucleoplasm, well separated from polytene chromosomes and the nuclear envelope, whereas the second subset represented trajectories observed in the vicinity of the nuclear envelope. A global fit to both subsets yielded four mobility components. Apart from an immobile fraction with *D*_1,ens_ = 0.046 ± 0.009 μm^2^/s and an unbound oligonucleotide fraction with *D*_4,ens_ = 4.4 ± 0.5 μm^2^/s, two fractions of intermediate mobility, *D*_2,ens_ = 0.26 ± 0.01 μm^2^/s and *D*_3,ens_ = 1.37 ± 0.05 μm^2^/s, were found, presumably representing mRNA diffusion in the nucleoplasm(9)(Supplementary Figure S11, Supplementary Table S3). The fraction of immobile and slowly diffusing particles increased significantly toward the nuclear envelope. These diffusion coefficients are smaller than expected theoretically for free diffusion of the particles with a diameter of approximately 50 nm, pointing toward transient interactions between the mRNA particles and fibrogranular clusters in the chromatin-free volume of the nucleus (9,34).

**Figure 4.**
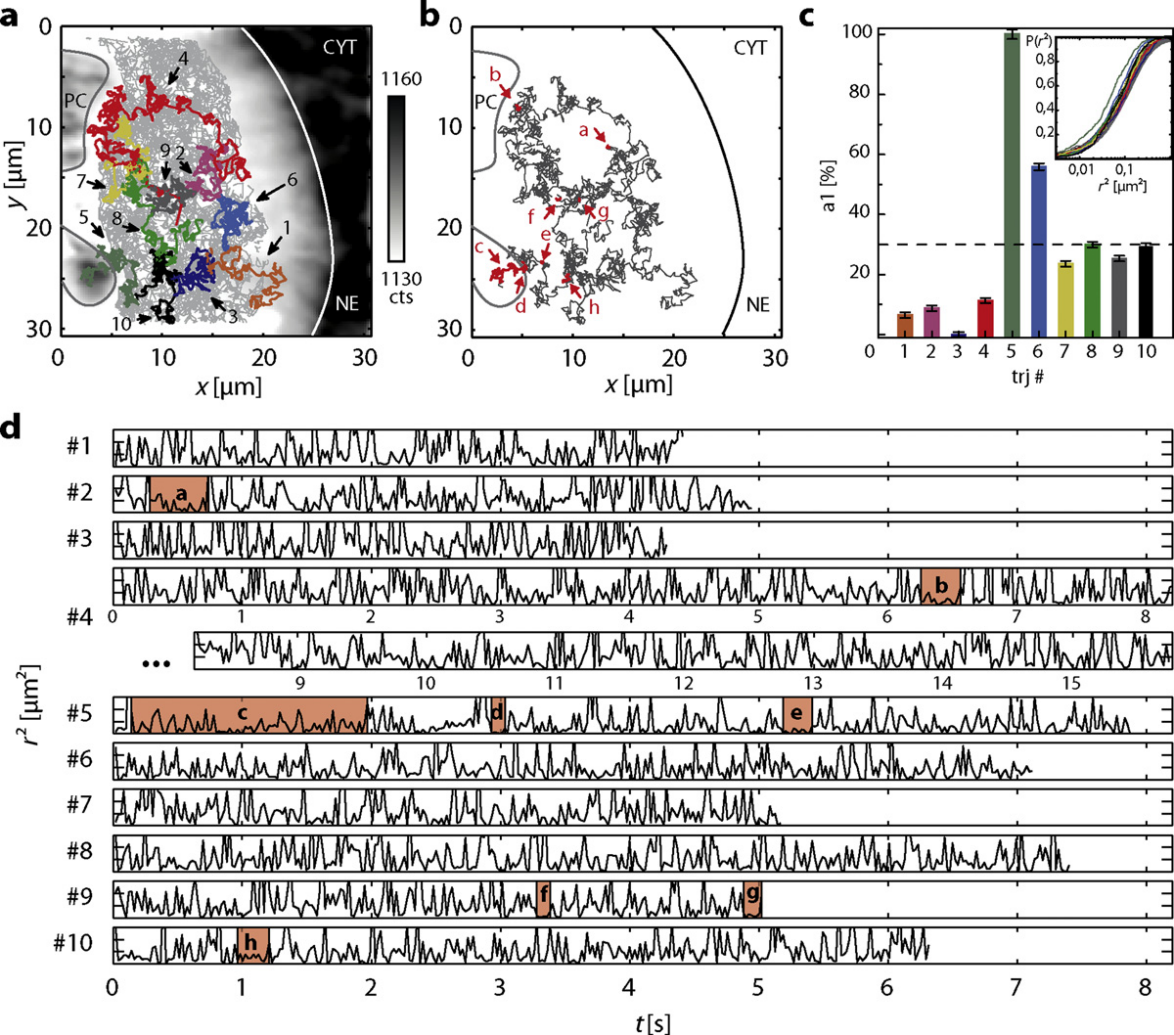
3D tracking of mRNPs in the nucleoplasm. (**a**) Ten trajectories comprising more than 200 localizations were found in a data set recorded in a single nucleus (Supplementary Figure S11). The gray line indicates a polytene chromosome (PC), the white line the nuclear envelope (NE) separating the nucleoplasm from the cytoplasm (CYT). (**b, d**) In the 10 long trajectories, eight dwell times in a state of reduced mobility were identified throughout the nucleoplasm (Supplementary Table S5). One of these dwell times (**c**) coincided with the part of trajectory #5 overlapping with the PC. (**c**) The cumulative jump distance distributions of the 10 trajectories (inset) were globally fitted with a bimodal distribution yielding *D*_1_ = 0.85 ± 0.01 μm^2^/s and *D*_2_ = 1.69 ± 0.01 μm^2^/s. A fit to the pooled data of all trajectories yielded a relative fraction of *a*_1_ = 30.0 ± 0.9% for the slower fraction. For the individual trajectories, *a*_1_ ranged from 0% (#3) to 100% (#5) (Supplementary Table S4). Apart from trajectory #5 located very close to a PC, no relation between *a*_1_ and position within the nucleoplasm was observed.

The data set contained a number of extremely long trajectories obtained by feedback tracking, which were used for a more detailed analysis. Ten trajectories comprised more than 200 localizations each (Figure 4a). The longest observation of an individual particle consisted of 769 localizations over a range of 10 μm in each spatial dimension with a total duration of 15.8 s at 20.5 ms temporal resolution (Supplementary Table S4, Supplementary Videos S4 and S5). On average, only 265 photons were detected per signal. The high number of localizations per trajectory enabled a comparative mobility analysis between the individual particles. In this small subset of long trajectories, only two mobility fractions with diffusion coefficients *D*_1_ = 0.85 ± 0.01 μm^2^/s and *D*_2_ = 1.69 ± 0.01 μm^2^/s (Figure 4c) were found by global fitting to the cumulative jump distance distributions of each trajectory (35). Notably, the relative contribution of the slower component ranged from 0 to 100% whereas a fit to the pooled data of these 10 trajectories yielded an average value of 30.0 ± 0.9% (Figure 4b, Supplementary Table S4). A global search for significant dwell times (*P‘*′ < 0.05) in a state of lower mobility across all possible threshold values *r*_lim_ yielded eight short phases with an unexpected absence of particle displacements larger than *r*_lim_ scattered throughout the nucleus (Figure 4b and d, Supplementary Table S5). One of the trajectories (#5) overlapped with a polytene chromosome. Strikingly, a long phase of reduced overall mobility could be identified in the respective segment of the trajectory (Figure 4b and d). This observation confirms the validity of the analysis, because it is known that the dense packing of polytene chromosomes strongly hinders diffusion of larger particles (9). All other dwell times were located in the nucleoplasm, well separated from both, the polytene chromosomes and the nuclear envelope. This substantiates previous findings suggesting discontinuous motion of mRNA particles in the same system discussed in detail in previous studies (9,34). However, the small number of localizations in trajectories limited to the static axial detection range hitherto prohibited to further discern the mobility characteristics and identify the discontinuous motion directly in isolated trajectories.

### mRNA tracking at the nuclear envelope

**Figure 5.**
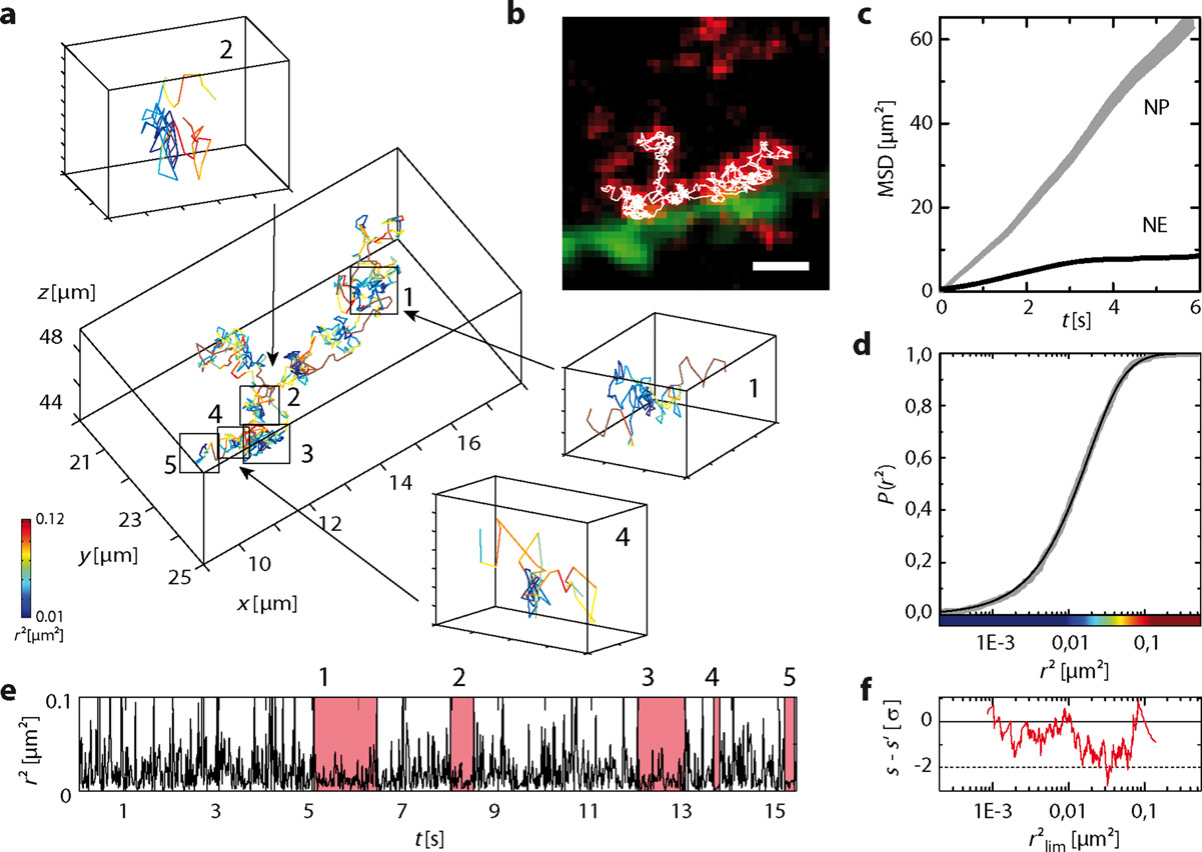
mRNA particle traveling along the nuclear envelope. (**a**) 3D representation of the full trajectory and parts of the trajectory exhibiting phases of reduced mobility (1–5) highlighted in (e) (Supplementary Table S5). (**b**) Overlay of nuclear envelope (green), maximum intensity projection of mRNA signal (red) and trajectory data (white) over a time course of 15.4 s (Supplementary Video S6). Scale bar, 2 μm. (**c**) Mean square displacement over time for trajectory #4 in Figure 4 (NP) and the trajectory presented in this figure (NE). The particle observed at the nuclear envelope exhibits a strongly reduced mobility. (**d**) Cumulative jump distance distribution and fit yielding two mobility fractions with *D*_1_ = 0.27 ± 0.01 μm^2^/s (93.7 ± 0.7%) and *D*_2_ = 1.61 ± 0.24 μm^2^/s (6.3 ± 0.7%). (**e**) Squared displacement as a function of time with phases of significantly reduced mobility indicated. (**f**) Difference between experimentally observed transition frequency *s* across a threshold *r*^2^_lim_ and expectation value *s*′ for random behavior in units of *σ_s’_*. The particularly low values indicate the presence of distinct mobility states.

The reduced overall particle mobility in the vicinity of the nuclear envelope found in the ensemble analysis was also reflected in individual feedback tracking trajectories. In Figure 5, an exemplary trajectory of 933 localizations acquired at a temporal resolution of 16.5 ms is displayed. Two mobility components *D*_1_ = 0.27 ± 0.01 μm^2^/s (93.7 ± 0.7%) and *D*_2_ = 1.61 ± 0.24 μm^2^/s (6.3 ± 0.7%) were obtained from a fit to the cumulative jump distance distribution *P*(*r^2^*) (Figure 5d), in agreement with results obtained from the ensemble analysis (Supplementary Table S4). Except for a short period of ∼2.5 s within the 15.4 s total observation time, the respective particle appeared to travel mostly along the nuclear envelope within a distance of less than 1.5 μm (Supplementary Video S6). The highly elongated shape of the trajectory (36) as well as the shape of the MSD curve (Figure 5c) suggest a 2D diffusion along the inner leaflet of the nuclear envelope. Such trajectories of mRNA particles gliding along the nuclear envelope, though much shorter, have been observed previously (37).

The high number of localizations for this individual particle again enabled a search for dwell times in distinct mobility states. Five significant phases of reduced mobility could be identified in close proximity to the nuclear envelope (Figure 5a and e, Supplementary Table S5). These more frequent dwell times might result from aborted nuclear export events, during which the mRNA particle probes a nuclear pore complex but is released again into the nucleoplasm. While isolated events of this kind have been reported previously (38), feedback tracking allows for the first time the observation of single mRNA particles apparently sampling several different nuclear pores sequentially. Such a behavior would also explain the apparent gliding of mRNA particles along the nuclear envelope.

Retention of the particle in states of either high or low mobility was additionally confirmed by an analysis of the transition frequency between particle displacements with *r*^2^(*t*) smaller or larger than a threshold value *r*^2^_lim_. For 0.01 μm^2^ < *r*^2^_lim_ < 0.08 μm^2^, the observed transition frequency *s* was more than one standard deviation *σ_s′_* smaller than the expectation value *s’*′ for a sequence of jump distances randomly drawn from the experimentally observed distribution *P*(*r*^2^) (Figure 5f). At *r*^2^_lim_ = 0.032 μm^2^, the deviation was larger than 2*σ_s′_* (*P’*′ < 0.023), indicating transient interactions between the particle and structures within the nucleus, which lead to dwell times in a state of low mobility of longer duration than expected in a random trajectory.

### 28S rRNA tracking

**Figure 6.**
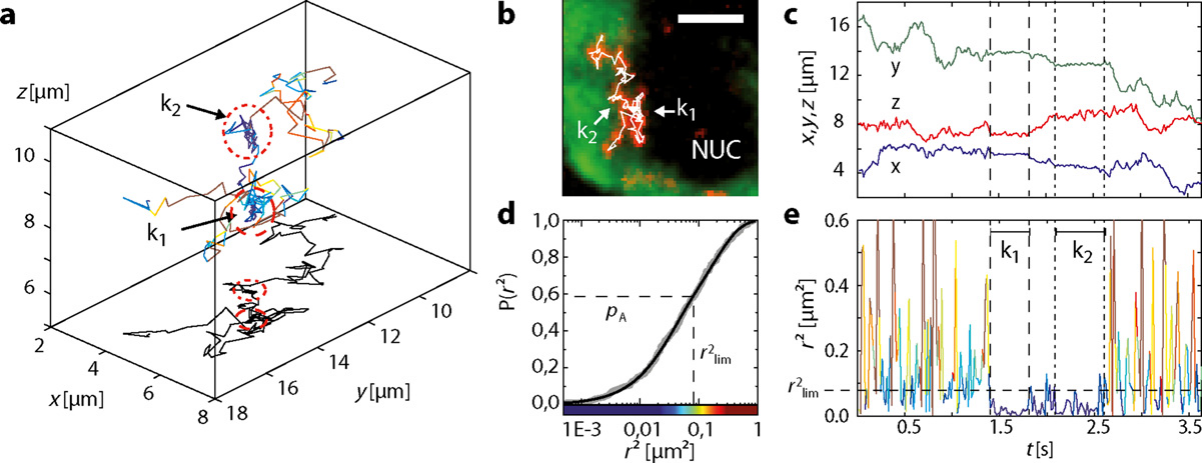
3D tracking of a ribosomal RNA particle. (**a**) Full 3D rRNA trajectory color-coded for particle displacement and *xy* projection of the trajectory (black) (Supplementary Video S7). See (d) for color-coding. 221 localizations at 16.5 ms temporal resolution were obtained. Interaction sites were marked by dashed circles. (**b**) Overlay of the trajectory with the NTF2-AF546 signal. NTF2 was enriched in the nucleoplasm (green), but excluded from the nucleolus (NUC). Maximum intensity projection (red) and trajectory data (white) of the rRNA particle. Scale bar, 5 μm. (**c**) Two transient binding events at the edge of the nucleolus were observable in the time course of the *x, y* and *z* coordinate. (**d**) A fit to the cumulative jump distance distribution yielded a bimodal distribution with *D*_1_ = 0.41 ± 0.01 μm^2^/s (40 ± 1%) and *D*_2_ = 2.87 ± 0.04 μm^2^/s (60 ± 1%). (**e**) Time course of the squared displacement *r*^2^(*t*) color-coded according to the jump distances. Dwell times *k*_1_ and *k*_2_ below the threshold *r*^2^_lim_ were evaluated.

For the first time our 3D tracking approach allowed to observe and analyze the complex dynamics of rRNA particles (rRNPs). Oligonucleotides targeted against the 28S subunit of the rRNA were microinjected into nuclei of *C. tentans* salivary gland cells. Labeled rRNPs exhibited a broad spectrum of mobilities with faster diffusion in the nucleoplasm than BR2.1 mRNA particles due to their smaller size and at the same time more distinct immobilization events discernible already before analysis of the trajectories. Mobile particles were found throughout the entire nucleus. However, in the nucleolus where rRNA is synthesized, particles were enriched and mostly immobile. An exemplary single rRNA trajectory is shown in Figure 6. Initially, the particle diffused through the nucleoplasm (Supplementary Video S7). At the border of the nucleolus, transient interactions led to repeated immobilization of the particle as evident from the temporal sequence of squared displacements *r*^2^(*t*) (Figure 6e). A fit to the cumulative jump distance distribution *P*(*r*^2^) yielded mobility fractions with diffusion constants *D*_1_ = 0.41 ± 0.01 μm^2^/s (40 ± 1%) and *D*_2_ = 2.87 ± 0.04 μm^2^/s (60 ± 1%).

The high number of 221 jump distances contained in this individual trajectory allowed for the identification of two highly significant dwell times in an immobile state. Retention times lasting *k*_1_ = 26 and *k*_2_ = 28 frames with *P′*-values *P′*(*k*_1_) = 5.2 × 10^−5^ and *P′*(*k*_2_) = 1.7 × 10^−5^, respectively were found at the edge of the nucleolus (Figure 6a, c and e). In this case, feedback tracking enabled the detection of repeated interactions between the rRNAP and the nucleolus. The axial separation between the interaction sites would have prevented both events to be recorded in a single trajectory in an experiment with a static detection plane. Notably, the virtually complete immobilization of the particle was not reflected in the diffusion coefficients recovered from the jump distance distribution.

## DISCUSSION

With the method presented here, it is for the first time possible to apply feedback tracking strategies to biomolecules labeled with only a single dye molecule, both, *in vitro* and *in vivo*, and even within large living multicellular specimens. Trajectories contain full spatial information and feedback tracking enables particle observations exceeding the axial detection range of 1 μm by an order of magnitude or more.

By adding a second and third dye molecule to the label, trajectory duration and SNR were further increased. While we relied on antisense oligonucleotides for *in vivo* tracking experiments, site-specific labeling with up to four cysteine-reactive dye molecules is readily achieved by adding a tetracysteine tag to the N- or C-terminus of a protein of interest (39). With trajectories comprising hundreds of localizations at hand, we can begin to investigate mobility patterns underlying biological processes on the basis of individual molecules. It should be noted that in contrast to previous feedback tracking approaches based on small image fields (17) or yielding 3D coordinates only for selected particles (11), the data sets presented here still contain the traditional ensembles of thousands of shorter trajectories (Supplementary Figure S12). Feedback tracking was used to enrich conventional single particle tracking data sets by a number of exceptionally long trajectories. 3D tracking techniques with a larger axial detection range hold the potential to produce similar data sets without a feedback mechanism, but lack the photon efficiency of our system (13). With the data at hand, it became possible to relate mobility fractions deduced from the statistical analysis of ensemble data sets to the behavior of individual particles. Based on trajectories comprising hundreds of localizations each, it is feasible to directly observe different mobility states, measure the times spent in these and determine transition frequencies between different states. Thus, the detailed analysis of individual trajectories can facilitate the assignment of mobility components found in the ensemble analysis to specific states of the particles under investigation. Effortless acquisition of reference images further supports the interpretation of single molecule trajectories.

The fast axial localization approach presented here might be of interest for real-time localization microscopy techniques, in which thousands of particles need to be localized in fractions of a second (40). Due to the relative simplicity of the calculations involved in the localization procedure, these tasks might even be executed on the FPGA chip of modern sCMOS cameras (41). Other localization algorithms like maximum likelihood estimators (42,43) have been shown to achieve higher and even optimal localization precision. Using such approaches, it might be possible to further improve the photon efficiency of feedback tracking if faster implementations of the algorithms or improved computer hardware become available. Our setup might further benefit from replacing the cylindrical lens with a deformable mirror capable of further optimizing the PSF shape for 3D localization (44). Furthermore, our approach can readily be combined with other illumination schemes like simple epi-fluorescence microscopes and optical sectioning by inclined (45) or reflected light sheet illumination (46). It can be anticipated that in smaller, adherent cells 3D single molecule tracking would be feasible for especially long times due to the lower fluorescence background. Progress in the field of stabilized or self-healing fluorescent dyes could further extend the scope of feedback tracking (47).

We employed the new tracking approach to examine mRNA and rRNA dynamics within living cell nuclei. Oligonucleotides carrying three organic dye molecules were synthesized to achieve highly specific labeling, increase SNR and enable the use of minimal illumination intensities. The number of localizations for individual molecules was augmented by more than one order of magnitude as compared to measurements with feedback tracking deactivated. The high number of localizations per trajectory obtained by 3D feedback tracking enabled RNA trafficking in the nucleus to be studied on the basis of individual particles rather than large ensembles of thousands of short trajectories. Previous experiments had already suggested a discontinuous motion of BR2.1-mRNA particles in the nucleoplasm of living *C. tentans* salivary gland cells (9,34), presumably caused by transient binding to intranuclear fibrogranular clusters. In those studies, however, the evidence for the discontinuous motion was only indirect. Taking into account the temporal sequence of the particle displacements in long individual trajectories in addition to their probability distribution now allowed for the first time the direct observation and unambiguous identification of transitions between states of different mobility. Furthermore, we examined here for the first time the dynamics of rRNA in the nuclear interior. Generally, 28S rRNPs diffused considerably faster than BR2.1-mRNA particles with maximal diffusion coefficients of 2.9 and 1.4 μm^2^/s, respectively. In stark contrast to the BR2.1-mRNA particles, 28S rRNAPs were immobilized completely and repeatedly rather than just reduced in their mobility. Such immobilizations were preferentially observed within nucleoli. We suspect that these immobilizations are directly connected to various steps of ribosomal biogenesis.

In general, extending the observation time for biomolecules carrying a single small emitter can be very instrumental in studying transient particle interaction, aggregation or self-organization phenomena. Dual color detection can be used to track a substrate in one channel while detecting binding events in a separate channel simultaneously. The method presented here obviates the need to immobilize one of the interaction partners on a glass surface or hold it in place by other means (19) and thus enables biomolecular interactions to be studied *in vivo*. We also foresee great potential for the technology presented here in tracking subdiffraction sized objects like vesicles (48) or viruses (49) and studying trafficking and transport phenomena on a single particle basis in larger specimen like 3D cell cultures or even entire model organisms.

## AVAILABILITY

Source code and instructions on how to use the source code are available for download at http://www.chemie.uni-bonn.de/pctc/kubitscheck/downloads.

## SUPPLEMENTARY DATA

Supplementary Data are available at NAR online.

SUPPLEMENTARY DATA
