# Supplementary material for: Direct observation of mobility state transitions in RNA trajectories by sensitive single molecule feedback tracking
Source: Nucleic Acids Res. 2014 Nov 20;43(2):e14. doi: 10.1093/nar/gku1194 (PMC4333372; doi:10.1093/nar/gku1194)
Supplement: SUPPLEMENTARY DATA [file supp_43_2_e14__index.html]

Direct observation of mobility state transitions in RNA trajectories by sensitive single molecule feedback tracking — Direct observation of mobility state transitions in RNA trajectories by sensitive single molecule feedback tracking — SUPPLEMENTARY DATA 

# Direct observation of mobility state transitions in RNA trajectories by sensitive single molecule feedback tracking

## SUPPLEMENTARY DATA

**Files in this Data Supplement:**

- SUPPLEMENTARY DATA
- SUPPLEMENTARY DATA
- SUPPLEMENTARY DATA
- SUPPLEMENTARY DATA
- SUPPLEMENTARY DATA
- SUPPLEMENTARY DATA
- SUPPLEMENTARY DATA
- SUPPLEMENTARY DATA
