# Supplementary material for: Direct observation of mobility state transitions in RNA trajectories by sensitive single molecule feedback tracking
Source: Nucleic Acids Res. 2014 Nov 20;43(2):e14. doi: 10.1093/nar/gku1194 (PMC4333372; doi:10.1093/nar/gku1194)
Supplement: SUPPLEMENTARY DATA [file supp_gku1194_nar-02683-met-g-2014-File008.pdf]

Direct observation of mobility state transitions in RNA trajectories by sensitive single molecule feedback tracking

Jan-Hendrik Spille, Tim Kaminski, Katharina Scherer, Jennifer S. Rinne, Alexander Heckel and Ulrich Kubitscheck

Supplementary information

Supplementary Fig. 1: Regular and astigmatic PSF........ 2

Supplementary Fig. 2: Astigmatic PSF in a calibration sample and in a biological specimen..... 2

Supplementary Fig. 3: DLL execution speed and localization precision........ 21

Supplementary Fig. 4: Flow diagram of the real-time tracking DLL 22

Supplementary Fig. 5: PSF measures and linearized calibration curves........ 5

Supplementary Fig. 6: Piezo step response time........ 5

Supplementary Fig. 7: High speed tracking........ 6

Supplementary Fig. 8: High speed tracking of small fluorescent beads........ 6

Supplementary Fig. 9: Single step photobleaching........ 7

Supplementary Fig. 10: Tracking mRNA carrying a single fluorophore........ 7

Supplementary Fig. 11: Spatial variation of mRNA mobility 8

Supplementary Fig. 12: Trajectory length distribution........ 8

Supplementary Table 1: Parameters passed to DLL.. 9

Supplementary Table 2: Mass spectrometry of labeled and unlabeled oligonucleotides. 9

Supplementary Table 3: Global fit results for mRNA trajectories.. 9

Supplementary Table 4: Global fit results for 10 individual, long trajectories 10

Supplementary Table 5: Significant dwell times found in mRNA trajectories.......................... 10

Supplementary Note 1: Real-time tracking DLL description 15

Supplementary Note 2: Oligonucleotide sequences.. 17

Supplementary Videos 18

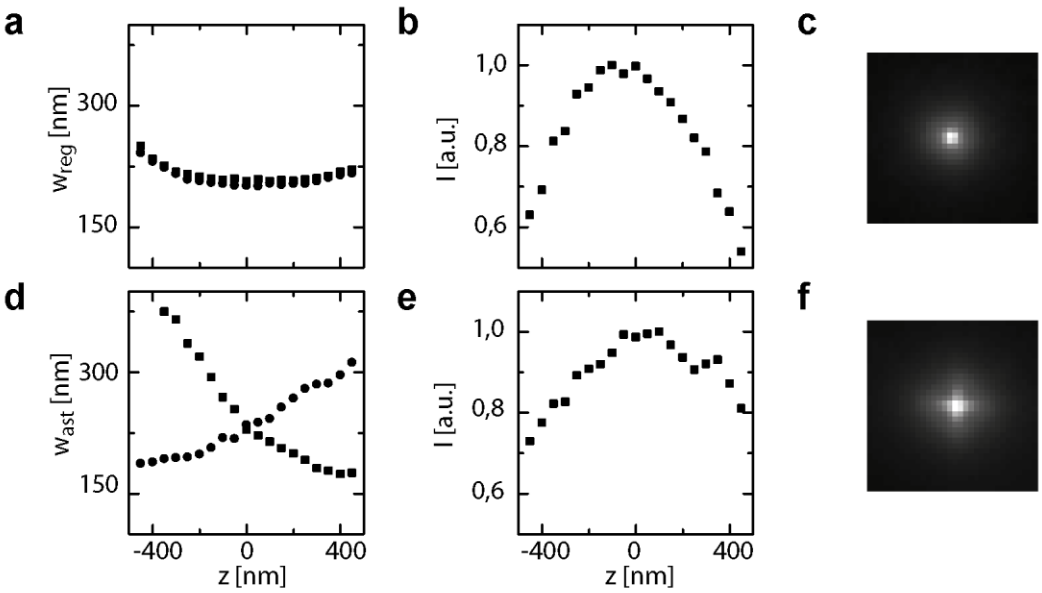

Supplementary Figure 1: Regular and astigmatic PSF. (a) Width of the regular PSF versus axial position (x - squares, y - circles) and (b) normalized amplitude as determined by fitting to a 2D Gaussian peak. (c) Sum projection of a z stack covering 1 µm axial range. Image field (7.9 µm)². (d) – (f) accordingly for the astigmatic PSF. Since only small distortions were introduced, reference images of continuous structures could be acquired with the cylindrical lens in place.

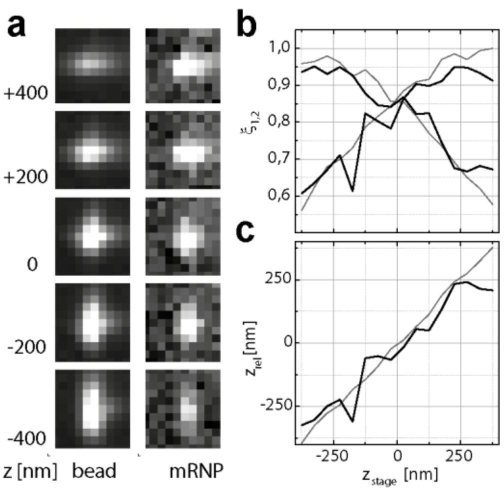

Supplementary Figure 2: Astigmatic PSF in a calibration sample and in a biological specimen. (a) Images of the PSF acquired with the emitter at different axial positions

relative to the focal plane. Comparison of the images of a fluorescent bead (∅ 200 nm) embedded in agarose and of a labeled mRNP bound to the nuclear envelope in a *C. tentans* salivary gland cell nucleus. (b) Normalized covariance values and (c) derived axial coordinate versus stage position (gray: bead, black: mRNP). The linear stage motion was reproduced for both samples.

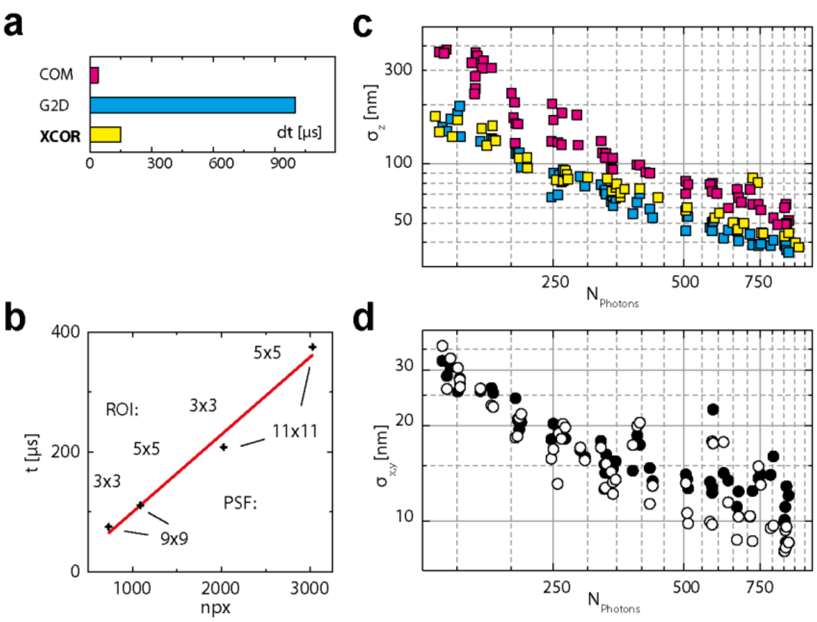

Supplementary Figure 3: DLL execution speed and localization precision. (a) DLL execution times for different localization algorithms. COM: 1st and 2nd moment calculation, 9x9 pixel subimage, 35 µs. G2D: Least squares fit of 2D Gaussian peak, 9x9 pixel subimage, 900 - >1700 µs. XCOR: Normalized covariance calculation. Template 9x9 pxl, executed for 5x5 pixel subimage. 75 – 370 µs. SNR = 8. (b) XCOR execution time as a function of the number of pixels involved (npx = 2 * number of pixels in PSF template * number of pixels in subimage). Results from a simulation of 10000 repeated evaluations on experimental data. (c) Axial localization precision as a function of the number of photons in a signal for the three different localization approaches experimentally determined using 200 nm fluorescent beads in 1.5% agarose. (d) Lateral localization precision (x – open circles, y – full circles) obtained from the G2D results.

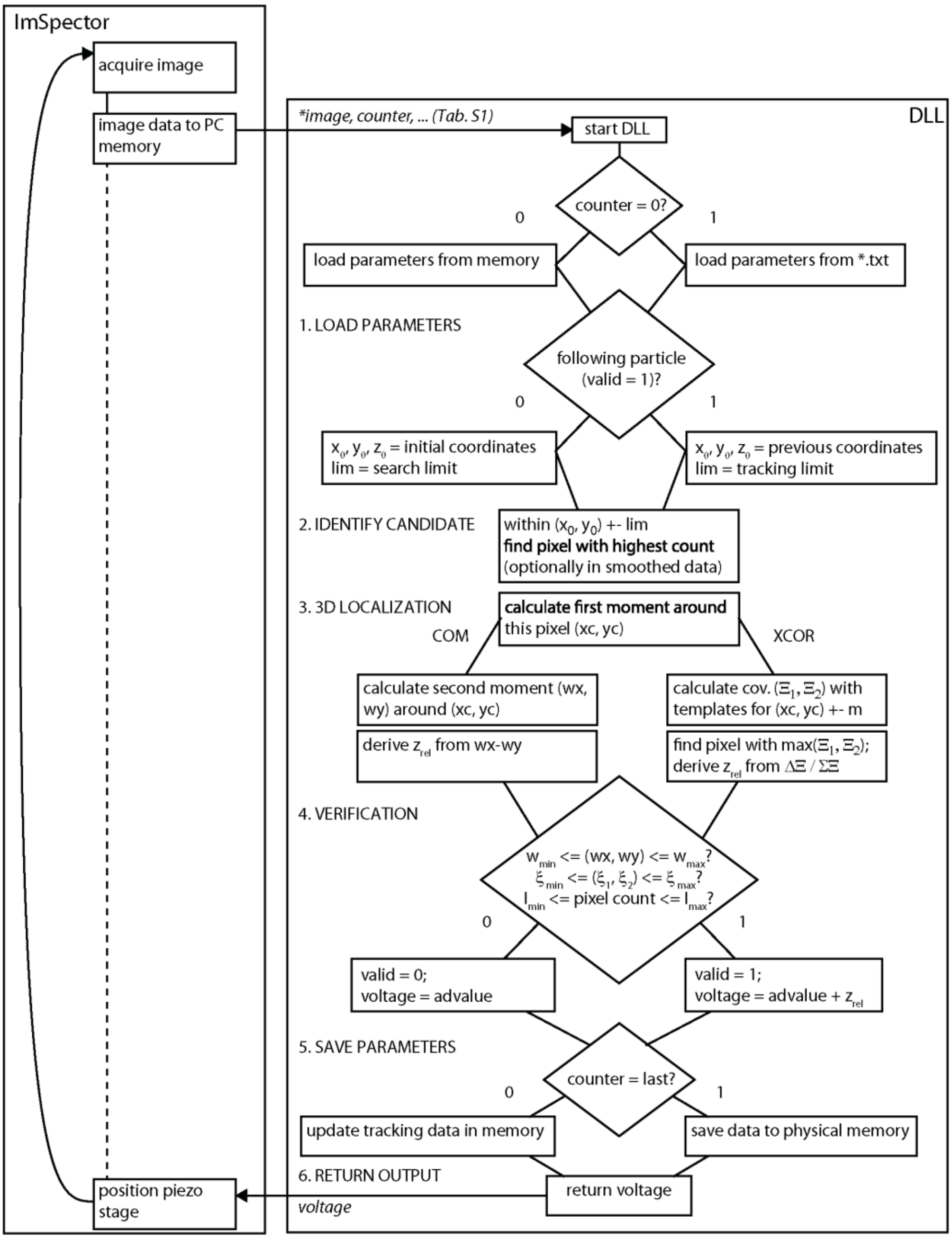

Supplementary Figure 4: Flow diagram of the real-time tracking DLL and ImSpector interface.

See Supplementary Table 1 for a list of parameters passed to the DLL.

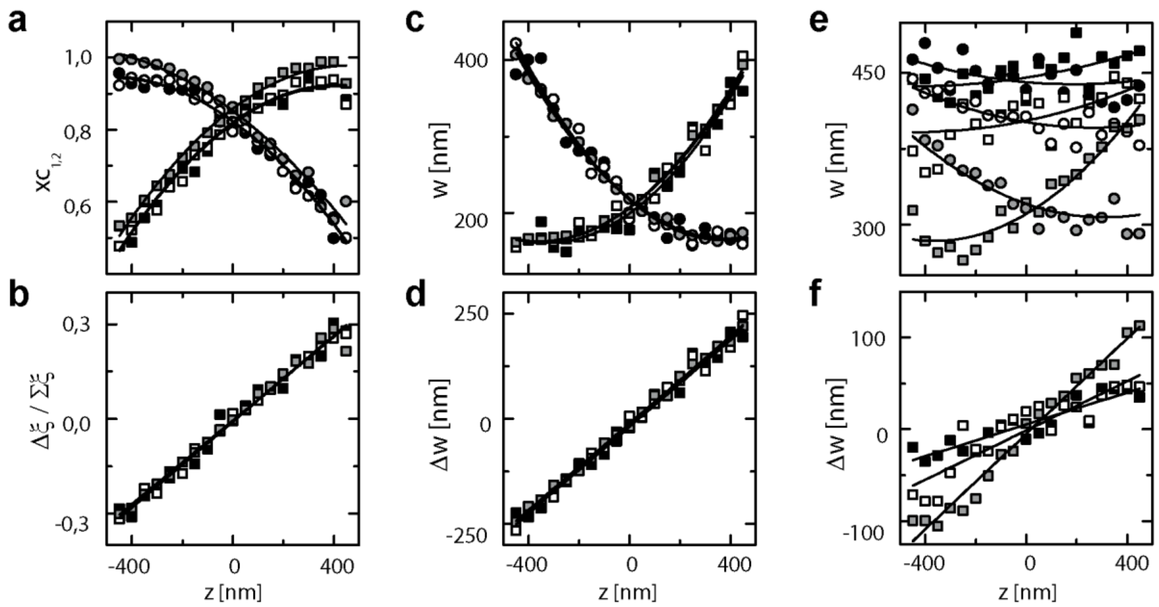

Supplementary Figure 5: PSF measures and linearized calibration curves for axial localization for SNR = 6 (black symbols), SNR=8 (white symbols), SNR=15 (grey symbols). SNR conditions simulated by adding appropriate Poisson noise to high SNR (~400) experimental data. (a) Normalized covariance values for PSF templates from below (circles) and above (squares) the focal plane. (b) Axial localization metric according to (eq. 1). The metric is obviously not dependent on the SNR. (c) PSF width along x (squares) and y (circles) as determined by fitting of a 2D Gaussian peak. (d) Δw = $w_x - w_y$ yields a linear calibration metric, which is again not dependent on SNR. (e) Second moment of the intensity distribution along x (squares) and y (circles). f) Δw still yields a linear metric for the axial localization, but the slope is dependent on the SNR.

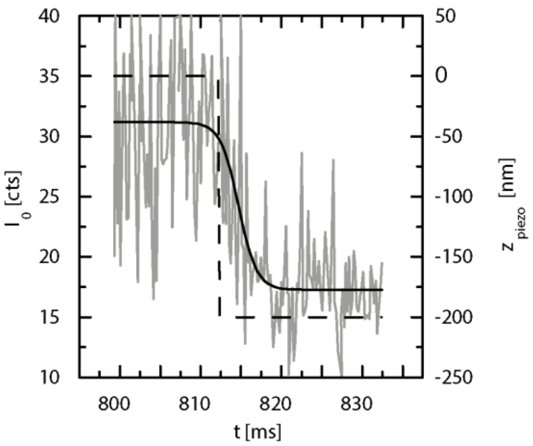

Supplementary Figure 6: Piezo step response time. The sample stage was axially displaced by 200 nm (black dashed line), resulting in a drop in peak amplitude in the signal recorded from an immobilized fluorescent bead imaged at 5.1 kHz frame rate by reading out 36 lines of an Orca Flash 4.0 camera (Hamamatsu). The drop in intensity was fitted by a logistic decay function with a time constant of 1.12 ± 0.48 ms.

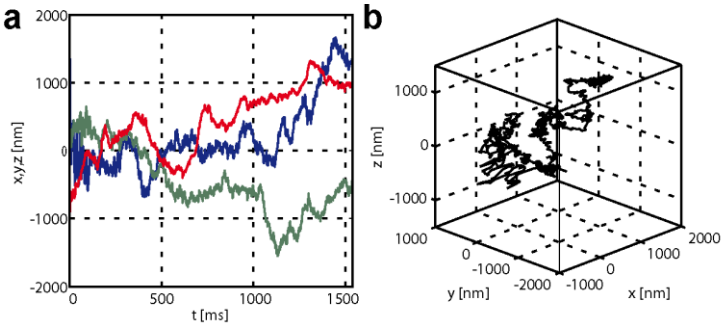

Supplementary Figure 7: High speed tracking of fluorescent particles in aqueous solution at a frame rate of 892 Hz using a pco.edge camera (Supplementary Video 1). a) Time course of x (blue), y (green) and z (red) coordinate and b) 3D reconstruction of the trajectory. Localizations were obtained at a temporal resolution of 1.12 ms. The piezo stage can be expected to lag behind particle motion at this imaging frequency.

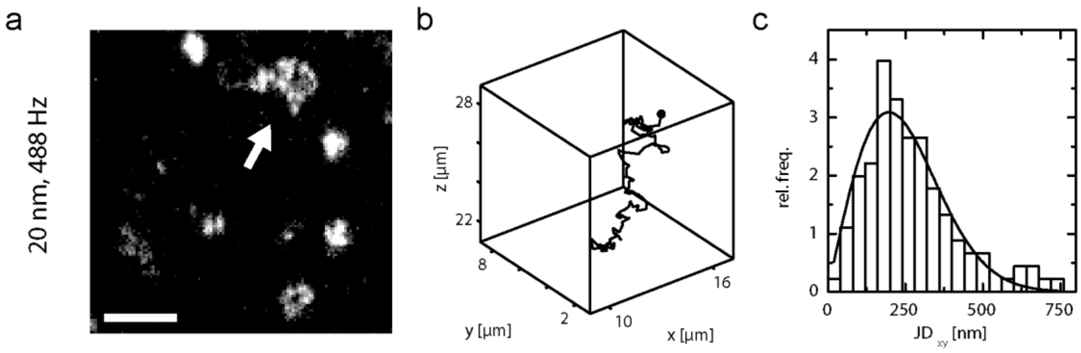

Supplementary Figure 8: High speed tracking of small fluorescent beads (FluoroSphere 0.02 µm crimson, Invitrogen) in aqueous solution at 488 Hz (Andor Ixon DU860 EMCCD camera). a) Maximum intensity projection of 114 frames acquired at a temporal resolution of 2.05 ms. A 20 nm bead was tracked with the feedback algorithm (arrow). Scale bar 5 µm. b) 3D reconstruction of the trajectory covering an axial range of more than 5 µm. c) Jump distance histogram and fit yielding a diffusion coefficient of $D = 9.4 \pm 0.8$ µm²/s.

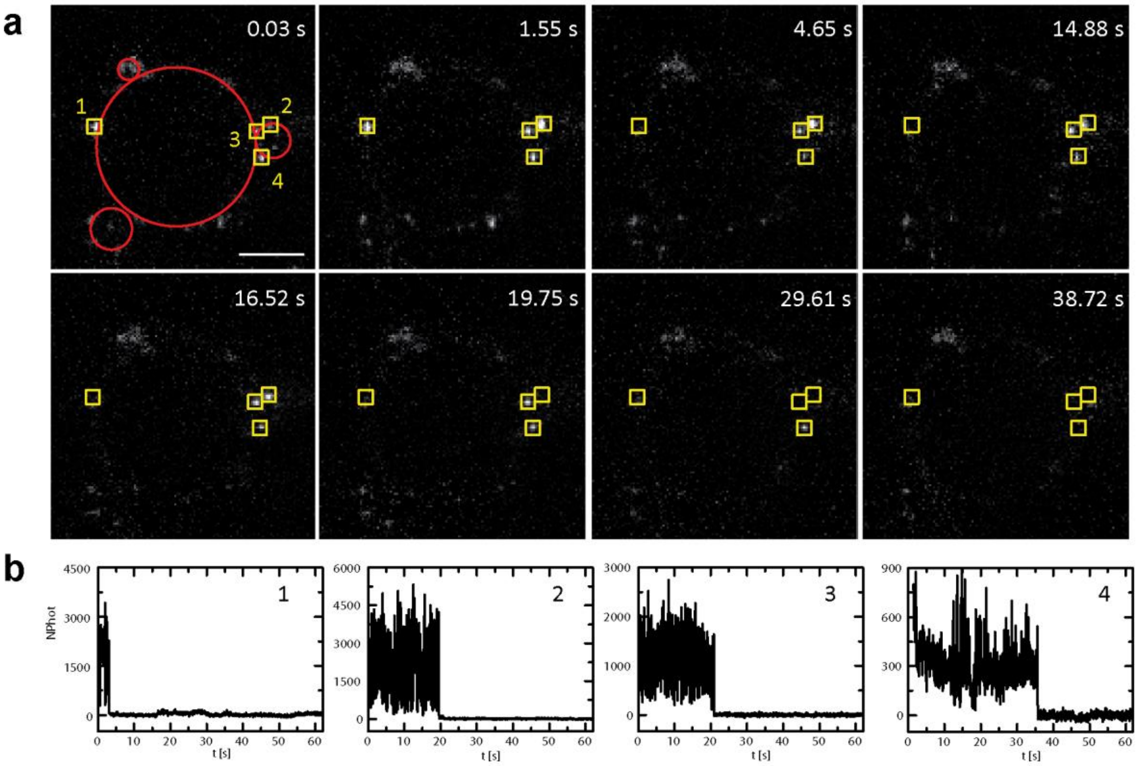

Supplementary Figure 9: Single step photobleaching of ATTO647- labeled DPPE in a highly viscous GUV membrane (90 mol% DPPC, 10 mol% cholesterol). (a) Stills from an image sequence acquired at a frame rate of 32 Hz. Red circles indicate the position of vesicles, yellow squares indicate positions of fluorescent molecules. (b) The time course of the number of photons per signal above background within the subimages of 11x11 pixels marked by the yellow squares indicates single step photobleaching as expected for lipids carrying a single dye molecule. An average of $N_{tot} = 4.7 * 10^5$ photons detected per emitter (n = 71) was found. Similar photon counts were achieved for mobile particles (Fig. 3). Scale bar 20 µm.

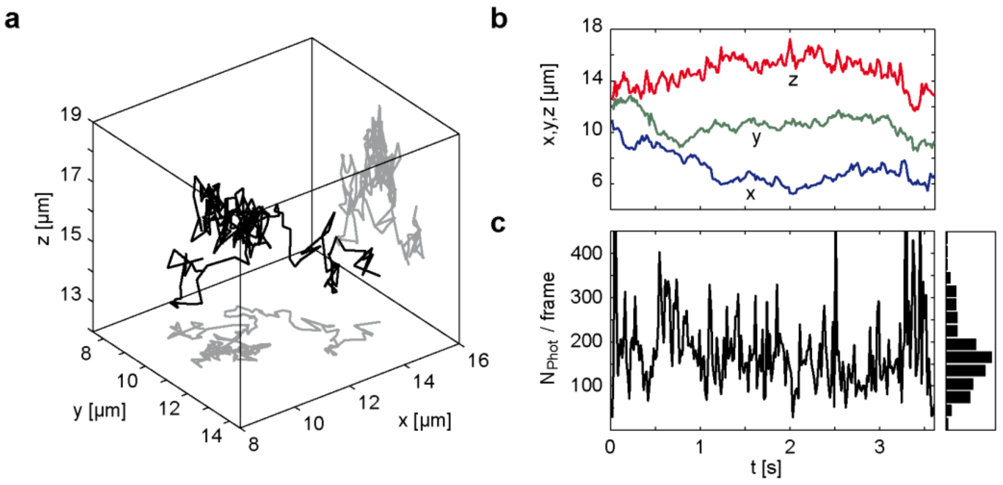

Supplementary Figure 10: Tracking mRNA carrying a single fluorophore. Exemplary trajectory covering 3.6 s over an axial range of more than 5 µm. (a) 3D representation of the trajectory. (b) x (blue), y (green) and z (red) position as a function of time. (c) On average, 177 photons per frame were detected from the single particle.

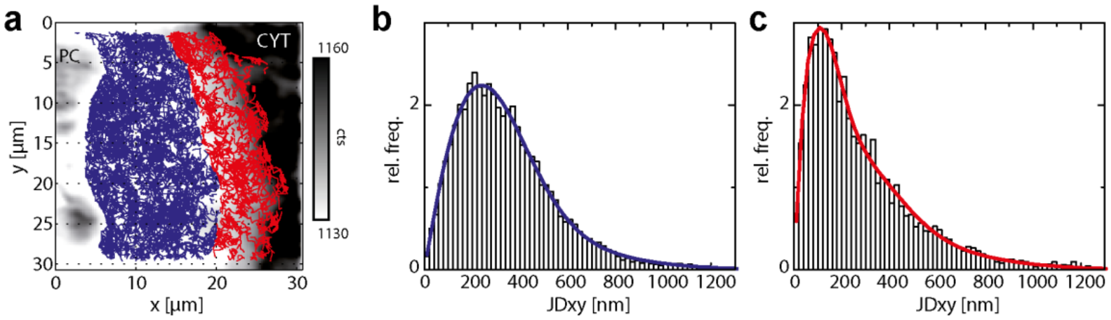

Supplementary Figure 11: Spatial variation of mRNA mobility in a *C. tentans* salivary gland cell nucleus. (a) Trajectories were spatially filtered to obtain one dataset (blue) characterizing particle motion in the nucleoplasm (NP) at a distance to both, polytene chromosomes (PC) and the nuclear envelope (NE), and one dataset due to particles moving in the vicinity of the nuclear envelope (red). Polytene chromosomes (PC) and cytoplasm (CYT) could be readily identified by the higher level of background noise as compared to the nucleoplasm. Trajectories were plotted on top of an average projection of the entire image sequence of 40000 frames. (b) Lateral jump distance distribution of the

nucleoplasmic (blue trajectories in a) and (c) the trajectories near the NE (red trajectories in a). Both histograms were fitted with the same set of diffusion coefficients (Supplementary Table 3). Towards the nuclear envelope, the fraction of slower moving particles increased.

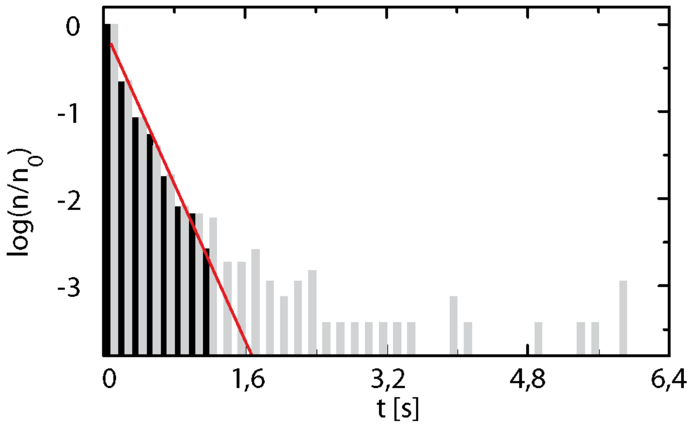

Supplementary Figure 12: Trajectory length distribution. Normalized values for a dataset with (gray) and without (black) feedback tracking obtained from the same sample. Activating feedback tracking does not change the length distribution of short trajectories comprising the vast majority of both datasets but adds trajectories with exceptionally high numbers of localizations. The red line indicates an exponential decay curve with time constant τ = 180 ms describing the relative frequency of short trajectories in both datasets. The duration of the longest trajectories (10 – 20s) matches the decay constant of photobleaching experiments conducted on immobile particles.

type **parameter**	description
*unsigned short** **image**	pointer to image data memory address
int **width**	width of image in pixels
int **height**	height of image in pixels
*double*** **profiles**	pointer to memory address of preallocated array profiles
*float*** **timestamp**	pointer to memory address of preallocated array timestamp
int **profileLen**	length of arrays, number of frames in experiment
double **advalue**	actual current voltage of piezo stage controller
double **davalue**	current goal voltage of piezo stage controller
unsigned int **counter**	counter for current image number within experiment
*bool** **imageChange**	indicates if new image data was written to memory

Supplementary Table 1: Parameters passed to DLL. *,** indicate pointer type variable and array.

**Oligonucleotide**	**Calc. mass [Da]**	**Mass found [Da]**
28S rRNA oligo	8765.8	8766.3
28S rRNA oligo-3xAtto647	10490.2	10491.5
BR2.1 3x oligo	10270.0	10271.3
BR2.1 oligo-3xAtto647N	12165.7	12166.3

Supplementary Table 2: Mass spectrometry of labeled and unlabeled oligonucleotides

region	N_{trj}	N_{disp}	a_1 [%]	a_2 [%]	a_3 [%]	a_4 [%]
all	1243	21533	0.7 ± 0.4	11.6 ± 0.8	66.2 ± 3.2	21.4 ± 3.3
nucleoplasm	760	14877	0.0 ± 0.3	1.8 ± 0.8	76.9 ± 3.4	21.9 ± 3.7
vicinity of nuclear envelope	483	6656	3.5 ± 0.7	34.0 ± 0.9	43.1 ± 2.8	19.3 ± 2.6

Supplementary Table 3: Global fit results for entire trajectory ensemble and subpopulations representing different regions within the nucleus. Relative fractions of the four mobility components D_{1ens} = 0.046 ± 0.009 µm²/s, D_{2ens} = 0.26 ± 0.01 µm²/s, D_{3ens} = 1.37 ± 0.05 µm²/s, D_{4ens} = 4.4 ± 0.5 µm²/s. N_{trj}: number of trajectories, N_{disp}: number of displacements per trajectory.

Trajectory #	N_{disp}	Δt [s]	N_{phot} [10^5]	a_1 [%]
1	216	4.4	0.6	6.5 ± 0.9
2	242	4.9	0.5	8.7 ± 0.9
3	210	4.3	0.5	0.0 ± 0.9
4	769	15.8	2.0	11.3 ± 0.8
5	385	7.8	1.4	100.0 ± 1.6
6	348	7.1	0.7	55.9 ± 1.1
7	253	5.1	0.5	29.9 ± 1.0
8	362	7.4	0.7	23.6 ± 0.9
9	246	5.0	0.6	25.3 ± 1.0
10	309	6.3	1.0	29.4 ± 1.0
all	3340	-	-	30.0 ± 0.9

Supplementary Table 4: Global fit results for 10 individual, long trajectories observed within the nucleoplasm. Mobility components: D_1 = 0.85 ± 0.01 µm²/s, D_2 = 1.69 ± 0.01 µm²/s. N_{disp}: number of displacements per trajectory, Δt: trajectory duration, N_{phot}: total number of photons detected from the particle, a_1: relative fraction of the slower mobility component in the trajectory.

Event	Trajectory #	r^2_{lim} [µm²]	p	Δt [s]
a	2	0.1512	0.034	0.451
b	4	0.1012	0.027	0.308
c	5	0.1991	0.014	1.825
d	5	0.0107	0.040	0.103
e	5	0.0436	0.047	0.226
f	9	0.0166	0.005	0.103
g	9	0.0429	0.045	0.144
h	10	0.0805	0.044	0.246
1	NE	0.0420	0.002	1.180
2	NE	0.0298	0.048	0.512
3	NE	0.0459	0.040	1.023
4	NE	0.0050	0.026	0.116
5	NE	0.0114	0.018	0.215

Supplementary Table 5: Significant dwell times and threshold values found in mRNA trajectories. r^2_{lim}: detection threshold, p: significance p-value according to eq. 4, Δt: duration of low mobility phase. a-h refer to trajectories in the nucleoplasm (Fig. 4), 1-5 were found in a single trajectory close to the nuclear envelope (Fig. 5).

Supplementary Note 1: Real-time tracking DLL description

Real-time image analysis and tracking code was implemented in terms of a DLL written in C++. The DLL was called immediately after an image frame had been transferred to PC memory. Parameters passed to the DLL are listed in Supplementary Table 1. Pre-allocated arrays were used to store tracking parameters, PSF templates and tracking results during the experiments. The general outline of the DLL is presented in Supplementary Fig. 4 and the source code as well as accompanying files and documentation is provided in Supplementary Material 1. During the first call to the DLL in an experiment, tracking parameters and PSF templates were loaded from ASCII files and stored in the previously mentioned arrays. Particle tracking consisted of six steps:

1. Loading parameters from an ASCII file or PC memory.
2. Candidate identification by detection of an intensity peak in a subimage around either initial, user-supplied coordinates or a previous particle localization. The size of the subimage was (i) small while following a particle to avoid trajectory confusion or (ii) large while searching for the first localization of a trajectory to increase real-time tracking duty cycle.
3. Centroid calculation for lateral localization and either second moment or normalized covariance calculation for determination of axial position relative to focal plane.
4. Particle verification based on peak intensity and either peak width or covariance values.
5. Storing tracking results and parameters in PC memory or, at the end of the experiment, in an ASCII file.
6. Return axial coordinate of the particle being tracked in terms of a piezo stage voltage.

To achieve robust tracking especially at low SNR, the algorithm may not be too sensitive on the one hand to avoid interpreting background noise fluctuations as signals resulting from fluorescence emission. On the other hand, occasional large axial particle displacements as well as fluctuations in the photon detection rate must be tolerated. Best results were obtained by leaving particle detection thresholds moderately high (e.g. minimum covariance value 0.35 - 0.45) and at the same time allowing for gaps in trajectories during real-time tracking by waiting for a particle to reappear within n_{wait} = 2-5 frames after the algorithm had lost it. This enabled recovering long trajectories using more sensitive particle detection parameters during post-processing without generating excessive amounts of false positive localizations during real-time tracking. If a trajectory was not continued after n_{wait} frames, the stage was set to proceed at its initial “home” position. At the end of an experiment, all arrays and tracking parameters were saved to ASCII files for further evaluation.

Commented source code for the tracking DLL, auxiliary files for setting parameters and providing template information as well as a PDF document with instructions on how to use the source code to set up a tracking experiment are available for download at http://www.chemie.uni-bonn.de/pctc/kubitscheck/downloads .

Supplementary Note 2: Oligonucleotide sequences

28S rRNA oligo:

5'- **H_2N-linker**-CAU UCG AAU AUU UGC **dT(NH_2)**AC UAC CAC CAA GAU CUG-**(NH_2)-linker**-3'

BR2.1 3x oligo:

5'-**H_2N-linker**-CUU GGC **dT(NH_2)**UG C**dT(NH_2)**G UGU **dT(NH_2)**UG CUU GG**dT(NH_2)** UUG C-**(NH_2)-linker**-3'

BR2.1 1x oligo:

5'-**H_2N-linker**- ACU UGG CUU GCU GUG UUU GCU UGG UUU GCU-3'

Supplementary Videos

Videos presented in the supplementary material were assembled using MATLAB and ImageJ. Two types of videos were created:

1. To present tracking data, raw image data were overlaid with trajectory information obtained from the post-processing analyis (colored lines connecting localizations), a timestamp indicating the time since the start of the experiment in milliseconds and an indicator of the current stage position. A square plotted on top of the image data indicates the subimage, in which the particle was searched for during tracking.
2. For a schematic representation of the tracking data, 3D plots of the trajectories were overlaid with surface renderings of reference structures (e.g., nuclear envelope or reconstructed vesicle surface). For the representation of time series, the current particle localization is indicated by a red sphere.

Video 1: High frequency tracking

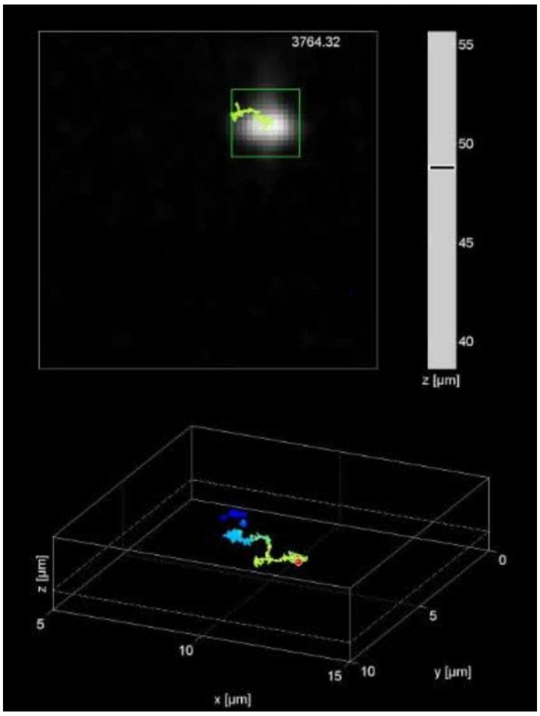

Fluorescent particle tracked in aqueous buffer. Overlay of raw data, real-time tracking information and trajectory data and corresponding 3D trajectory plot. 892.5 Hz original frame rate displayed at 50 Hz. Image field (10.56 µm)².

Video 2: Lipid tracking

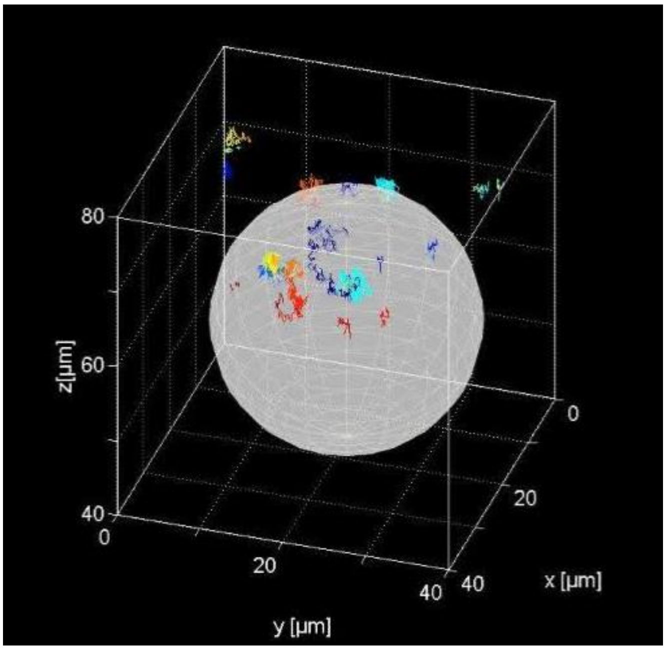

POPE lipids carrying a single ATTO647 dye were tracked on the surface of a giant unilamellar vesicle at an average of 130 detected photons per signal. 3D coordinates were determined for all lipids and the shape of the vesicle estimated by fitting a sphere to all localizations. Data were acquired and displayed at 63 Hz frame rate. Image field (40.96 µm)^2.

Video 3: mRNA tracking single fluorophore

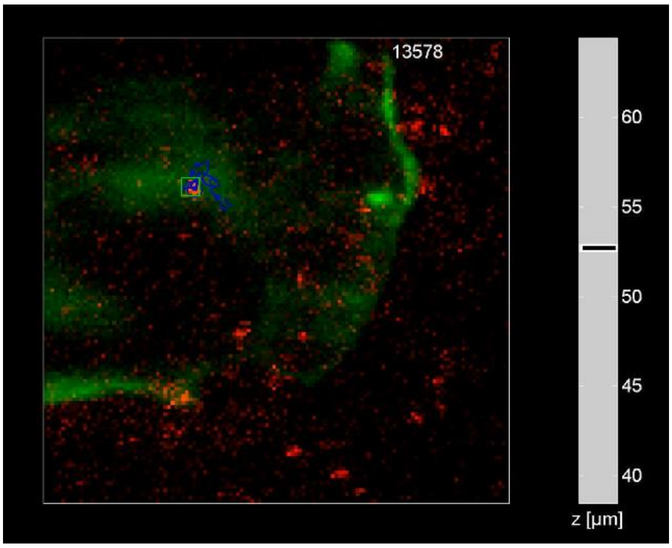

mRNA particle labeled with an oligonucleotide carrying a single Atto647 dye in the nucleoplasm (red). NTF2-AF546 was co-injected to stain the nuclear envelope (green). Single trajectory of 3.6 s duration tracked at 62.5 Hz displayed at 20 Hz. Image field (30.7 µm)^2.

Video 4: mRNA tracking nucleoplasm

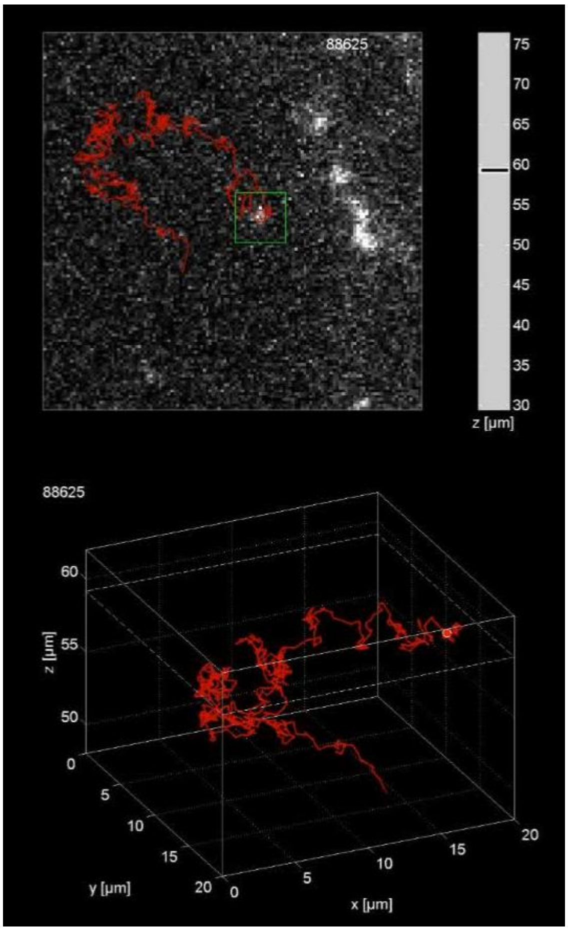

mRNA particle tracking in the nucleoplasm. 15.6 s trajectory raw data and tracking data overlay. 3D Plot of mRNA particle trajectory. Image field (30.72 µm)².

Video 5: mRNA tracking nucleoplasm trajectory reconstruction

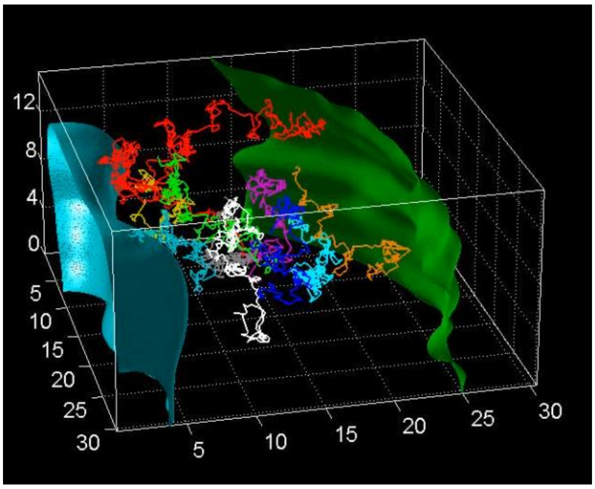

3D plot of 10 long mRNA particle trajectories and surface renderings of a polytene chromosome (cyan) and the nuclear envelope (green).

Video 6: mRNA tracking nuclear envelope

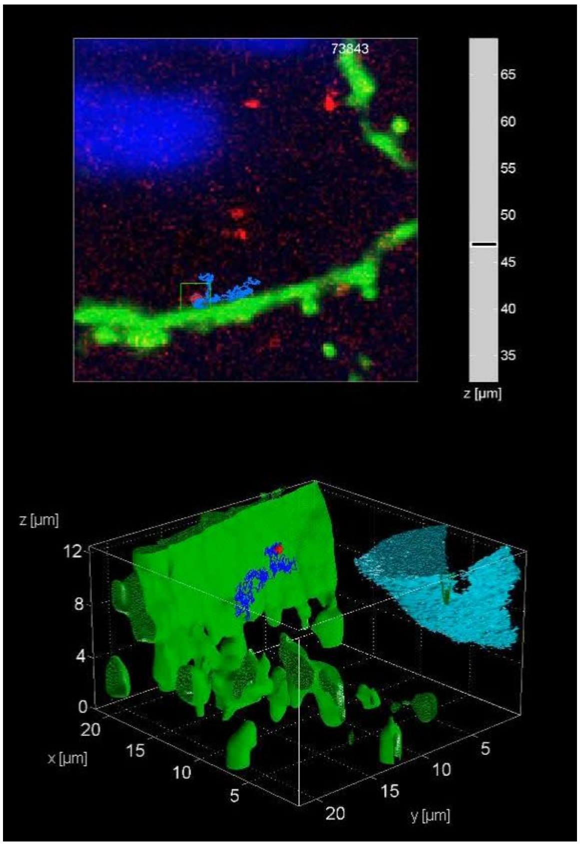

mRNA particle tracking at the nuclear envelope. Raw data overlayed with trajectory and reference information (polytene chromosome, cyan; nuclear envelope, green) and 3D rendering of reference structures and mRNA particle trajectory. Image field (30.72 µm)².

Video 7: rRNA tracking nucleolus

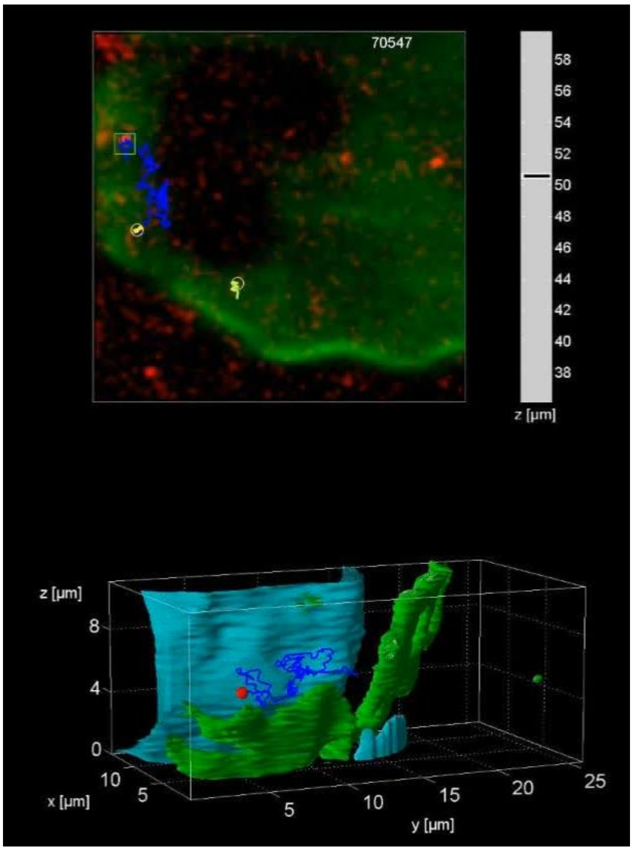

rRNA particle tracking at the nucleolus. Raw data overlayed with trajectory and reference information (nuclear envelope, green) and 3D rendering of reference structures (nuclear envelope, green; nucleolus, cyan). Nucleolus reconstructed from negative contrast in NTF2-AF546 channel (green), compare upper panel. Image field (30.72 µm)^2.
